# The Importance of Methyl-Branched Cuticular Hydrocarbons for Successful Host Recognition by the Larval Ectoparasitoid *Holepyris sylvanidis*

**DOI:** 10.1007/s10886-020-01227-w

**Published:** 2020-10-29

**Authors:** Sarah Awater-Salendo, Hartwig Schulz, Monika Hilker, Benjamin Fürstenau

**Affiliations:** 1 Institute for Ecological Chemistry, Plant Analysis and Stored Product Protection, Julius Kühn Institute, Federal Research Centre for Cultivated Plants, Königin-Luise-Str. 19, 14195 Berlin, Germany; 2 Dahlem Centre of Plant Science, Institute of Biology, Applied Zoology/Animal Ecology, Freie Universität Berlin, Haderslebener Str. 9, 12163 Berlin, Germany

**Keywords:** Bethylidae, GC-MS analysis, Host recognition, Methyl alkanes, Stored product pest, Tenebrionidae, *Tribolium*

## Abstract

Cuticular hydrocarbons (CHCs) of host insects are used by many parasitic wasps as contact kairomones for host location and recognition. As the chemical composition of CHCs varies from species to species, the CHC pattern represents a reliable indicator for parasitoids to discriminate host from non-host species. *Holepyris sylvanidis* is an ectoparasitoid of beetle larvae infesting stored products. Previous studies demonstrated that the larval CHC profile of the confused flour beetle, *Tribolium confusum*, comprises long chain linear and methyl-branched alkanes (methyl alkanes), which elicit trail following and host recognition in *H. sylvanidis*. Here we addressed the question, whether different behavioral responses of this parasitoid species to larvae of other beetle species are due to differences in the larval CHC pattern. Our study revealed that *H. sylvanidis* recognizes and accepts larvae of *T. confusum*, *T. castaneum* and *T. destructor* as hosts, whereas larvae of *Oryzaephilus surinamensis* were rejected. However, the latter species became attractive after applying a sample of *T. confusum* larval CHCs to solvent extracted larvae. Chemical analyses of the larval extracts revealed that CHC profiles of the *Tribolium* species were similar in their composition, while that of *O. surinamensis* differed qualitatively and quantitatively, i.e. methyl alkanes were present as minor components on the cuticle of all *Tribolium* larvae, but were absent in the *O. surinamensis* CHC profile. Furthermore, the parasitoid successfully recognized solvent extracted *T. confusum* larvae as hosts after they had been treated with a fraction of methyl alkanes. Our results show that methyl alkanes are needed for host recognition by *H. sylvanidis*.

**Supplementary Information:**

The online version of this article (10.1007/s10886-020-01227-w) contains supplementary material, which is available to authorized users.

## Introduction

Cuticular hydrocarbons (CHCs) are present on the surface of all insects and have several important functions, ranging from protection against desiccation to mediation of intra- and interspecific communication (Gibbs 1998; Ingleby 2015; Menzel et al. 2017; Otte et al. 2018). CHCs play a key role in the chemical communication of many insect species, serving as contact sex pheromones and kairomones for recognition of gender, caste, nest mates, mutualistic partners, and hosts (Howard and Blomquist 2005). The information conveyed by CHCs is based on various CHC types, which may be linear or methyl branched, saturated or unsaturated hydrocarbons with different chain lengths. The CHC patterns vary from species to species; some of these patterns are simple and contain only a few compounds, while others are composed of highly complex multi-component blends with up to 100 compounds (Blomquist and Bagnères 2010).

Despite the complexity of insect CHC profiles, many studies pointed out that in general only a small number of components are involved in the intra- and interspecific communication of insects. For some species, single compounds of a specific CHC blend are sufficient to elicit behavioral responses (Colazza et al. 2007; Guédot et al. 2009; Kühbandner et al. 2012b; Rutledge et al. 2009), whereas for others a combination of several CHCs is required (Sakata et al. 2017; Spikes et al. 2010; Sugeno et al. 2006; Würf et al. 2020). A few studies have demonstrated the behavioral activity of straight chain (linear) alkanes and alkenes (Colazza et al. 2007; Dani et al. 2005; Ginzel et al. 2003b). In many cases, however, methyl alkanes have turned out to be the behavior inducing cues (e.g. Guédot et al. 2009; Ginzel et al. 2003a; Lacey et al. 2008; Ruther et al. 2011; Sakata et al. 2017; Silk et al. 2011).

Several species-specific CHCs have already been identified to be used by parasitic wasps to discriminate between mating partners or host species (Ablard et al. 2012; Kühbandner et al. 2012b). For generalist parasitoids, it is assumed that their foraging behavior is mediated by general cues released by different hosts (Steidle and Van Loon 2003), but only little is known about chemical cues used by parasitoid species that attack a broader range of host species.

The polyphagous, cosmopolitan ectoparasitoid *Holepyris sylvanidis* (Bréthes 1913) (Hymenoptera: Bethylidae) has been described to parasitize larvae of various beetle taxa (i.e. Cucujidae, Silvanidae, Tenebrionidae) infesting processed plant products (Amante et al. 2017b; Evans 1969; Hagstrum and Subramanyam 2009). Adults and larvae of these beetles are major pests of stored grain or wheat flour and occur worldwide in warehouses and in the food-processing industry (Hertlein et al. 2011; Vassilakos et al. 2012).

In a previous study we showed that *H. sylvanidis* responds to larval CHCs of its preferred host, the confused flour beetle *Tribolium confusum* (Du Val 1863) (Coleoptera: Tenebrionidae). The parasitoid uses the CHCs of the host larvae for host finding at short range by following CHC trails laid by larvae on the substrate (Fürstenau and Hilker 2017). The CHC profiles of some other potential host species of *H. sylvanidis* have also been chemically analyzed in the past (Baker et al. 1978; Howard et al. 1995; Lockey 1978), but their influence on host recognition or host selection of parasitoids has not yet been examined. Therefore, it is still unknown, whether *H. sylvanidis* recognizes suitable host larvae by a CHC pattern, which different host species have in common.

To address this question, we selected three beetle species which have been described as potential host species of *H. sylvanidis*; (i) *T. confusum*, (ii) the red flour beetle *T. castaneum* (Herbst 1797) (Coleoptera: Tenebrionidae) and (iii) the sawtoothed grain beetle, *Oryzaephilus surinamensis* (Linnaeus, 1758) (Coleoptera: Silvanidae). In addition to the aforementioned study by Fürstenau and Hilker (2017), several previous studies have shown that *T. confusum* and the closely related species *T. castaneum* are accepted as hosts by *H. sylvanidis* (Amante et al. 2017a, 2018; Ahmed et al. 1997; Fürstenau et al. 2016). Because of co-occurrence of *H. sylvanidis* and *O. surinamensis* in storage facilities (e.g. Eliopoulos et al. 2002) this grain beetle has been also considered a potential host species (Amante et al. 2017b; Hagstrum and Subramanyam 2009). However, it is unknown as yet, whether *H. sylvanidis* indeed accepts this species as host and whether the parasitoid can successfully develop on this species. Moreover, the destructive flour beetle, *Tribolium destructor* (Uyttenboogaart 1934) (Coleoptera: Tenebrionidae), a distantly related species of *T. confusum* and *T. castaneum*, was also included as a fourth species in the investigation without ever being described as a host of *H. sylvanidis*. However, it is interesting to note that adults of all three *Tribolium* spp. release 1-pentadecene, a volatile compound, which is considered to be part of the flour beetle aggregation pheromone (Arnaud et al. 2002; Verheggen et al. 2007) and has also been demonstrated to attract *H. sylvanidis* (Fürstenau et al. 2016).

The aim of the present study was to answer the following questions: (i) Do *H. sylvanidis* females recognize and accept larvae of different stored product pest beetle species as hosts? (ii) Does the larval CHC profile of a successfully recognized host species differ from that of a non-host? (iii) Which compounds of the CHC profile of a potential host species act as contact cues for successful host recognition by *H. sylvanidis*?

We analyzed hexane extracts of larvae of the aforementioned four beetle species by gas chromatography coupled with mass spectrometry (GC-MS) and compared the CHC profiles. In order to find out, whether methyl alkanes are responsible for host recognition by *H. sylvanidis*, we isolated them from larval extracts of *T. confusum.* Subsequently, the behavioral responses of *H. sylvanidis* to live and dead larvae of the four beetle species, to crude larval extracts of a host or a non-host species and to a fraction of methyl alkanes of the *T. confusum* CHC profile were tested in contact bioassays.

## Methods and Materials

### Insects

The four stored-product pest beetles and potential host species of *H. sylvanidis*, *T. castaneum*, *T. confusum*, *T. destructor*, and *O. surinamensis* were reared in the laboratory of the Institute for Ecological Chemistry, Plant Analysis and Stored Product Protection (Julius Kühn Institute (JKI), Berlin, Germany). The host insects were reared in a climate chamber in permanent darkness at 25 ± 1 °C and 65 ± 5% RH.

The three analyzed *Tribolium* spp. were kept on the same feeding substrate, a mix of finely ground wheat grist (*Triticum aestivum* Linnaeus 1753) and wheat flour (Type 405, Kaufland, Neckarsulm, Germany, mix = 1:1), in 400 ml glass jars. Rearing of *T. confusum* was conducted as described by Fürstenau et al. (2016). The rearing protocol for *T. castaneum* and *T. destructor* was similar, but modified as follows: Two weeks after emergence from the pupal stage, 150 adults of *T. castaneum* and 50 adults of *T. destructor* were taken from the permanent rearing at the JKI and each placed in a glass jar filled with 150 ml of feeding substrate. The adults (males and females) were left in the jars for one week and could mate and oviposit during this time. Thereafter, they were separated from the feeding substrate by sieving (mesh size = 710 µm) and transferred to another jar filled with fresh feeding substrate. The egg-infested substrate was stored for subsequent larval development. Approximately five weeks after oviposition, *Tribolium* larvae reached the 4th instar level, the (most) preferred host stage for *H. sylvanidis*. For our rearing, we used adult beetles for at maximum two weeks before replacing them by freshly emerged adults from the permanent rearing.

*Oryzaephilus surinamensis* was reared on coarsely ground wheat grist. Fifty adults (males and females) were kept in a 400 ml glass jar. After mating and oviposition for one week, the adults were removed manually with forceps and placed in a jar filled with new feeding substrate. Beetles were used for at maximum three weeks before they were replaced by new (freshly emerged) ones for the further rearing. The substrate infested with *O. surinamensis* eggs was stored for subsequent larval development. In each jar we additionally placed a tissue paper to provide a good shelter for the latest instar larvae before pupation and to facilitate the removal of *O. surinamensis* larvae for following bioassays and chemical analysis. Larvae were removed approximately four weeks after oviposition when *O. surinamensis* larvae were 4th instars.

The parasitoid species *H. sylvanidis* was reared on *T. confusum* larvae in a climate chamber (25 ± 1 °C, 57 ± 5% RH). Previous studies had shown that experience with host or host-associated products (e.g. host exuviae or feces) just after emergence can induce behavioral changes in the parasitoid, resulting in reinforcement of inherited host preference (Barron 2001). Therefore, unexperienced (“naïve”) *H. sylvanidis* females were used in the host recognition bioassays to exclude any biased effects.

To obtain naïve *H. sylvanidis*, one- to seven-day-old females were collected from the permanent rearing at the JKI (Berlin, Germany) and placed individually with one conspecific male in a Petri dish (94 mm diam., 16 mm height). Each Petri dish was provided with approx. 50 *T. confusum* 4th − 5th instars as hosts and 1.8 g ground wheat grist as food for the host larvae. A honey drop was applied onto the inner surface of the Petri dish lid for carbohydrate nutrition. After having located a host larva, the *H. sylvanidis* female paralyzes the host and pulls it to a sheltered site prior to laying its egg onto the host larva. Therefore, five pipette tips (0.1–20 µl, Carl Roth, Karlsruhe, Germany) were offered in each Petri dish as hiding place for paralyzed host larvae. To prevent parasitoids from escaping, Petri dishes were sealed with parafilm. *Tribolium confusum* larvae were taken from our stock culture. The adult parasitoids could mate and oviposit onto the host larvae for one week. Thereafter, they were transferred to a new Petri dish to mate and oviposit for another week before they were replaced by newly emerged ones. Parasitized host larvae were separated from unparasitized ones and placed individually in a Petri dish (5.0 cm diam.). After pupation of the parasitoid larvae (approx. one week after oviposition) we gently removed host exuviae and larval feces of *T. confusum* with a brush. Thus, freshly emerging *H. sylvanidis* adults had no chance to experience chemical cues of their rearing host, *T. confusum.* Naïve *H. sylvanidis* adult progenies emerged four to six weeks after oviposition and could feed on a drop of honey. Newly emerged parasitoids were separated by sex. Unmated, naïve, one- to four-day-old females were used in bioassays, while older females and males were discarded.

### Preparation of Crude Larval Extracts from Potential Host Species

For chemical analyses of the CHC profiles of potential host species and subsequent contact bioassays, we prepared the following larval extracts: (1) crude extracts from 4th instar larvae of each species (*T. castaneum*, *T. confusum*, *T. destructor* and *O. surinamensis*) for GC-MS analysis, (2) crude extracts from 4th instar larvae of *T. confusum* and *O. surinamensis* for contact bioassays (*dummy test I*, treatments #7,8,11,12 in Table 1), (3) crude extracts from *T. confusum* 4th instar larvae for separation of *n*-alkanes from methyl branched ones and for contact bioassays testing the different CHC samples (*dummy test II*, treatment #15–16 in Table 1).

**Table 1:** Overview of contact bioassays for analyses of host recognition behavior by *Holepyris sylvanidis* females

Bioassay	*#*	Potential host species	State^a^	Treatment^b^	*N*^*c*^
A) Host recognition — Influence of beetle species	1	*T. castaneum*	L/D	untreated	36
	2	*T. confusum*	L/D	untreated	36
	3	*T. destructor*	L/D	untreated	36
	4	*O. surinamensis*	L/D	untreated	36
B) *Dummy test I *— Influence of host larval CHCs	5	*T. confusum*	D	untreated	30
	6	“	D	extracted^†^	30
	7	“	D	extracted^†^ + re-applied *O. sur*-larval extract^‡^	30
	8	“	D	extracted^†^ + re-applied *T. con*-larval extract^‡^	30
	9	*O. surinamensis*	D	untreated	30
	10	“	D	extracted^†^	30
	11	“	D	extracted^†^ + re-applied *O. sur*-larval extract^*^	30
	12	“	D	extracted^†^ + re-applied *T. con*-larval extract^*^	30
C) *Dummy test II* — Influence of host larval CHC fractions	13	*T. confusum*	D	untreated	40
	14	“	D	extracted^†^	40
	15	“	D	extracted^†^ + re-applied *T. con*-larval extract — **complete CHC profile**^**^**^	40
	16	“	D	extracted^†^ + re-applied **methyl-branched CHC-fraction**^**^^**^ of *T. con*-larval extract	40

^a^Live (**L**) or differntly treated dead (**D**) larvae were offered to parasitoids. Larvae were killed at -20 °C. Behavioral responses and searching time until successful host recognition were recorded for a period of 300 s

^b^Different treatments for dead larvae as test stimuli:

^†^To remove cuticular hydrocarbons, larvae were extracted for 10 min with *n*-hexane

^‡^2 µl (= ½ larval equivalent, LE) of *T. confusum* (*T. con*) or *O. surinamensis* (*O. sur*) crude larval extract were re-applied to extracted larvae

^*^1 µl (= ½ LE) of *T. con* or *O. sur* crude larval extracts were re-applied to extracted larvae

^^^2 µl (= ½ LE) of *T. con* larval extract were re-applied to extracted larvae before fractionation with 5 Å-molecular sieves

^^^^2 µl (= ½ LE) of methyl alkane fraction of *T. con* larval extract were re-applied to extracted larvae after fractionation with 5 Å-molecular sieves

^c^Number of replicates

A previous study by Fürstenau and Hilker (2017) demonstrated that the chemical composition of processed CHC extracts of *T. confusum*, which had been purified via solid-phase extraction, did not differ from crude extracts of the same beetle species. Therefore, we prepared and used only crude larval extracts of the aforementioned beetle species for our chemical analyses and bioassays.

1. Crude larval extracts of the four potential host beetle species for chemical analysis were prepared by a procedure slightly modified from that described by Fürstenau and Hilker (2017). Preliminary tests revealed that only small amounts of compounds can be extracted from the cuticle of *O. surinamensis* larvae. Therefore, crude extracts of *O. surinamensis* larvae (*N* = 20) were prepared by immersing 90 larvae in 120 µl *n*-hexane (analytical purification > 98%, VWR, Radnor, USA) for 10 min at ambient temperature. We concentrated the extract under a gentle stream of nitrogen and stored it at -20 °C for further analysis. The 4th instar larvae of the studied host species differed somewhat in size and weight. For example, larvae of *O. surinamensis* were smaller (approx. 4–5 mm) than those of *Tribolium* spp. (approx. 6–10 mm). To prepare crude larval extracts with comparable larval biomass per solvent, we calculated the mean weight of 10 samples with 90 *O. surinamensis* larvae each (= 59.03 mg ± 0.66). We took this value to determine the number of larvae to be used for the other beetle species (*T. confusum* = 25 larvae, *T. castaneum* = 28 larvae, *T. destructor* = 21 larvae). Thereafter, crude larval extracts of *Tribolium* spp. (*N* = 20 for each species) were prepared as described for those of *O. surinamensis*. To quantify the amount of host larval CHCs, extracts were re-dissolved in 50 µl *n*-hexane containing 1-eicosene as internal standard (IS, 10.4 ng µl^− 1^, Sigma-Aldrich, Taufkirchen, Germany).

(2) Crude larval extracts for contact bioassays were produced as follows: Ten larvae of *T. confusum* or of *O. surinamensis* were immersed in 100 µl *n*-hexane and removed after 10 min before the supernatant was dried under a gentle stream of nitrogen. For the re-application of CHCs on dead and extracted *T. confusum* larvae, the extracted CHCs were re-dissolved in 40 µl *n*-hexane (treatments #7–8 in Table 1). For re-application of CHCs on dead and extracted *O. surinamensis* larvae, the extracted CHCs were re-dissolved in 20 µl *n*-hexane (treatments #11–12 in Table 1). Since *T. confusum* larvae are larger and thicker than those of *O. surinamensis*, 2 µl of crude larval extract were required to uniformly impregnate a *T. confusum* larva. In contrast, applying 1 µl was enough to evenly cover an *O. surinamensis* larva.

(3) Crude larval extracts of *T. confusum* for fractionation and further contact bioassays with different CHC samples were produced by following a method described by Bello et al. (2015). Approximately 2000 freshly killed *T. confusum* larvae were extracted in 5 ml *n*-hexane for 10 min. The supernatant was concentrated to ca. 1000 µl under a gentle stream of nitrogen. To purify the alkanes (both linear and methyl branched), we loaded the crude extract onto an isolute silica gel column (100 mg, Biotage, Uppsala, Sweden), which had been pre-conditioned by rinsing two times with 1 ml dichloromethane (> 99%, Merck, Darmstadt, Germany) and 1 ml *n*-hexane. The sample was eluted from the column by applying four times 1 ml *n*-hexane.
  From this eluate (purified CHCs), we took (i) 450 µl (≈ 200 LE of *T. confusum*) for subsequent contact bioassays and (ii) 100 µl (≈ 44 LE of *T. confusum*) for chemical analysis by GC-MS; both types of samples were stored at -20 °C.
  To separate the methyl alkanes from linear ones, the remaining eluate was transferred to a 25 ml vial and concentrated to dryness before being re-dissolved in 5 ml isooctane (Merck, Darmstadt, Germany). We added 100 mg of activated 5 Å-molecular sieves (Sigma-Aldrich, Taufkirchen, Germany) per mg of sample, while the vial was flushed with nitrogen for 5 min. To activate the molecular sieves, they had previously been dried in a muffle furnace at 300 °C for 15 h. In the airtight-sealed vial, the extract was then magnetically stirred at ambient temperature for 18 h. Thereafter, the supernatant containing isolated methyl alkanes was removed and filtered through a Whatman filter paper (9.0 cm diam.). Prior to storage of this supernatant for later contact bioassays, 100 µl (≈ 44 LE of *T. confusum*) were taken for GC-MS analysis. In total, we fractionated three samples of larval extracts (*N* = 3). For chemical analysis, we used 100 µl each of the supernatant containing isolated methyl alkanes and of the eluate containing purified CHCs (*N* = 3 per sample type). Under a gentle stream of nitrogen, we concentrated each sample to dryness before adding 50 µl *n*-hexane containing 1-eicosene (10.4 ng µl^− 1^) as IS.
  To test the influence of different structural groups of CHCs on the host recognition behavior of *H. sylvanidis*, we used purified CHCs (mixture of linear and methyl alkanes) as well as isolated methyl alkanes, which were obtained by separation from *T. confusum* crude larval extracts. For treatment #15 (Table 1), we concentrated 46 µl of each sample with purified CHCs (≈ 20 LE of *T. confusum*) under a gentle stream of nitrogen and dissolved each sample in 80 µl *n*-hexane. For treatment #16 (Table 1), we took 50 µl (≈ 20 LE of *T. confusum*) of each sample with isolated methyl alkanes and prepared the extracts as described for those in treatment #15.

### GC-MS Analysis of Crude Larval Extracts of Potential Host Species and Different CHC Samples of *T.confusum* Larvae

GC-MS analyses of crude larval extracts and different CHC samples of *T. confusum* larval extracts were performed on a GCT Premier – TOF Mass Spectrometer (Waters, Milford, USA) coupled to a GC System 7890A (Agilent Technologies, Waldbronn, Germany). One µl of each sample was injected in splitless mode, keeping the injector at 250 °C with helium as carrier gas (1 ml min^− 1^). The oven temperature program started at 40 °C, which was held for 4 min and increased then at 10 °C min^− 1^ to 300 °C. The final temperature was held for 20 min. Samples were separated on a 30 m HP-5MS capillary column (250 µm diam., 0.25 µm film thickness, Agilent JandW Scientific). After a solvent delay of 5 min, masses were scanned every 0.9 s with a range from 50 to 600 *m*/*z* (electronic impact [EI] ionization = 70 eV, source temperature = 230 °C).

For structure assignments of detected compounds, an authentic *n*-alkane standard (*n-*C7-*n*-C40, Sigma-Aldrich, Taufkirchen, Germany) was additionally injected. Linear alkanes were identified by comparing their mass spectra and the calculated retention indices (RIs) with those of the *n*-alkane standard. In contrast, no reference compounds of the methyl alkanes were available to us. For each compound, we tentatively determined the position of its methyl branching based on the characteristic mass spectrometric fragmentation and the calculated RIs according to van den Dool and Kratz (1963). We further compared the RI and the fragmentation pattern with those published by Lockey (1978), Hebanowska et al. (1989, 1990), Howard et al. (1995), Spiewok et al. (2006), Geiselhardt et al. (2009), Svensson et al. (2014), Gerhardt et al. (2016), and Fürstenau and Hilker (2017).

Individual compounds were quantified relative to the peak area of the IS. CHC samples were standardized by calculating the mean amount of each compound (in ng) per one larval equivalent (LE).

### Contact Bioassay: Host Finding and Recognition Behavior

 To analyze the response of one-to four-day-old, naïve *H. sylvanidis* females to 4th instar (live and dead) larvae of (i) *T. confusum*, (ii) *T. castaneum*, (iii) *T. destructor* and (iv) *O. surinamensis*, we performed a series of different contact bioassays (Table 1). The bioassay methods were similar to those described by Fürstenau and Hilker (2017). The parasitoid behavior was observed in a test arena, which consisted of a circle (9.0 cm diam.) drawn on a sheet of white paper and covered by the lid of a plastic Petri dish (94 mm diam., 16 mm height). To avoid any external interference, the test arena was surrounded by a cardboard box (70.5 cm x 44.5 cm x 41 cm). For an even illumination, a strip of light-emitting diodes (λ = 625 nm, Barthelme GmbH & Co, Nürnberg, Germany) was located 5 cm above the box. All bioassays took place at 25 ± 1 °C. Live or differently treated dead larvae of each species were placed individually in the center of the test arena. Beetle larvae were killed by freezing at -20 °C for 2 h and allowed to warm to room temperature for 30 min prior to biotest start. In three different experimental set ups we tested the influence of the following host stimuli on the host recognition behavior of *H. sylvanidis* (Table 1):

A) untreated larvae of the previously mentioned beetle species
B) crude larval extracts (CHC profiles) of *T. confusum* or *O. surinamensis*, respectively, applied onto extracted larvae of these two species
C) different CHC samples of *T. confusum* larvae (including one sample of isolated methyl alkanes) applied onto extracted *T. confusum* larvae

Untreated as well as extracted larvae of the respective species were used as control treatments in bioassays B) and C).

All bioassays were prepared by placing the host stimulus (a single live or dead larva) in the center of the test arena. When a crude larval host extract was applied onto a test larva, the solvent could evaporate for 1 min prior to release of the parasitoid into the arena. A bioassay began by releasing a single *H. sylvanidis* female onto the circle drawn on the sheet of paper lining the arena. The release side of parasitoids rotated clockwise to avoid any biased results due to possible side preference. Each individual parasitoid was observed for max. 300 s. As described by Fürstenau and Hilker (2017), we recorded (i) whether the parasitoid located a host larva and showed host recognition behavior when encountering it and (ii) determined the searching time until successful host recognition. We defined successful host recognition as the moment when *H. sylvanidis* bends its abdomen around the larva to start paralyzing it. Once the parasitoid successfully found and recognized its host, the experiment was stopped. Test individuals, host stimuli, and the paper lining the arena were replaced by new ones after each run. The lid of the Petri dish was cleaned with a 70% ethanol solution (> 96%, Berkel AHK, Ludwigshafen, Germany). When a parasitoid rested more than 50% of the observation time - less than 5% (34 occasions) of all bioassays listed in Table 1 - the individual was not included in the statistical analysis and replaced by a new one, which showed active searching behavior. When live or dead larvae of *Tribolium* spp. and *O. surinamensis* were offered as potential hosts (bioassay “A”), each treatment was replicated 36 times (*N* = 36). When we studied the influence of crude larval extracts of *T. confusum* or *O. surinamensis* on host recognition of *H. sylvanidis* (bioassay “B”), we repeated each treatment 30 times (*N* = 30). When the influence of different CHC samples of *T. confusum* larvae on host recognition of *H. sylvanidis* were tested (bioassay “C”), each treatment was replicated 40 times (*N* = 40).

### Bioassay: Host Acceptance and Oviposition

To figure out whether the parasitoid accepts larvae of *Tribolium* spp. and *O. surinamensis* for oviposition, we conducted a further bioassay, which additionally allowed us to check the development of the parasitoid´s offspring on the beetle larvae. *Holepyris sylvanidis* deposits a single egg onto a host larva and only a single parasitoid larva can develop per host (Amante et al. 2017a).

A naïve, one-to four-day-old *H. sylvanidis* was offered one live 4th instar larva in a Petri dish (5.0 cm diam.) for a period of 24 h. The potential host larva was provided with 0.4 g finely ground wheat grist. Furthermore, one pipette tip (0.1–20 µl, Carl Roth, Karlsruhe, Germany) was offered as hiding place to the parasitoid since *H. sylvanidis* pulls paralyzed host larvae to a shelter site prior to oviposition. For each potential host species, we tested 40 female parasitoids on 40 host larvae (*N* = 40 per species). After the 24-hour-exposure time to the parasitoid, the parasitized larvae were transferred to a climate chamber (25 ± 1 °C, 57 ± 5% RH, permanent darkness) for further development. Larvae onto which the parasitoid had oviposited, were recognized by the parasitoid’s egg on the cuticle of the host larval. Unparasitized larvae were classified as not-accepted hosts. After four weeks, the number of emerging parasitoids per host species were counted (= successful host acceptance). Host larvae, which were not accepted as hosts and those on which *H. sylvanidis* larvae had not completed its development, were counted as failed host acceptance.

### Statistical Analysis

All statistical analysis were computed in “R”, version 3.6.1 (R Core Team 2019), except of the SIMPER analysis, which was performed in “PAST”, version 3.26 (Hammer et al. 2001).

For an across-beetle-species-comparison, we prepared data sets obtained by chemical analyses of the CHC profiles as follows. For some peaks, the mass spectrum and RI indicated that several internally branched alkanes (branching at position 10, 11, 12, 13, 14 or 15) co-eluted. The RIs of these compounds differed slightly among the *Tribolium* species due to the different positions of the methyl branching. Therefore, we pooled these internally branched alkanes. Additionally, we only included compounds, which were present in more than 50% of all extracts of a beetle species. When one of the selected compounds was below the detection limit in some extracts of a beetle species, we handled the missing compound as follows. To avoid any bias in the subsequent statistical analysis, we generated a random peak for each missing value by the “rnorm()”-function in “R”. Since the missing compound had been detected in other extracts of a beetle species, we selected the smallest peak area, which this compound had in these extracts, as mean and calculated the standard deviation based on the four smallest peak areas. In total, 43 pseudo peaks were generated. Finally, we normalized all selected compounds by calculating the quantitative contribution of each compound to one LE of the beetle species.

To statistically compare the CHC patterns of the tested beetle species, we calculated relative amounts of detected compounds in 1 LE of each beetle species and summed up all amounts to 100%. Based on the Bray-Curtis dissimilarity, a one-way analysis of similarity (ANOSIM) was then performed with 99,999 random permutations using the package “vegan” (version 2.5-6, Oksanen et al. 2019) in “R”. The dissimilarity of groups is stated by the *R*-value, ranging between 0 and 1. An *R*-value close to 1 indicates a clear discrimination, whereas an *R*-value close to 0 indicates a high similarity (Clarke 1993). We also applied an analysis of similarity percentages (SIMPER) to identify compounds, which contribute the most (i) to the dissimilarity between the CHC profiles of *Tribolium* spp. or (ii) to the dissimilarity between the CHC profiles of *Tribolium* spp. and *O. surinamensis*. The differences among CHC profiles were visualized by performing a non-metric multidimensional scaling (NMDS) calculated on Bray-Curtis dissimilarity. The stress value associated with NMDS indicates how close the algorithm of NMDS fits to the used data set. A NDMS with a stress value < 0.1 indicates a good fit of the NMDS ordination or low data distortion (Clarke 1993; Dexter et al. 2018).

A comparison of the behavioral responses of *H. sylvanidis* to the offered different host stimuli was based on (i) the host recognition rate per potential host species/treatment and (ii) the mean searching time until successful host recognition of the offered larva. The host recognition rate was analysed by the test of equality of proportions followed by a pairwise comparison of proportions with *Bonferroni-Holm* correction (Newcombe 1998a, b). When the parasitoid’s response to dead, differently treated *O. surinamensis* larvae (treatments #9–12 in Table 1) was tested in contact bioassays, results were evaluated by *Fisher’s* exact test, followed by a pairwise comparison of proportions with *Bonferroni-Holm* correction. Since the *Shapiro-Wilk* test of normality revealed that the mean searching times required by the parasitoids were not normally distributed in all treatments, we applied the *Kruskal-Wallis* test for comparing the mean searching time of parasitoids exposed to different host stimuli. Thereafter, differences in the mean searching time between the different treatments were pairwise compared using *Wilcoxon* rank sum test with *Bonferroni-Holm* correction.

To analyze the host acceptance behavior of *H. sylvanidis*, we determined for each species the number of larvae successfully accepted as hosts and of those that were not. We recorded “successful host acceptance”, when parasitoid offspring emerged from the host and “failed host acceptance”, when host larvae were left unparasitized or when parasitoid larvae could not successfully develop inside the host. Finally, the proportions of successful and failed host acceptance were analyzed by the test of equality of proportions followed by a *Bonferroni-Holm* corrected pairwise comparison of proportions across the beetle species.

## Results

### Comparison of Larval CHC Profiles of Possible Host Species

**Fig. 1:**
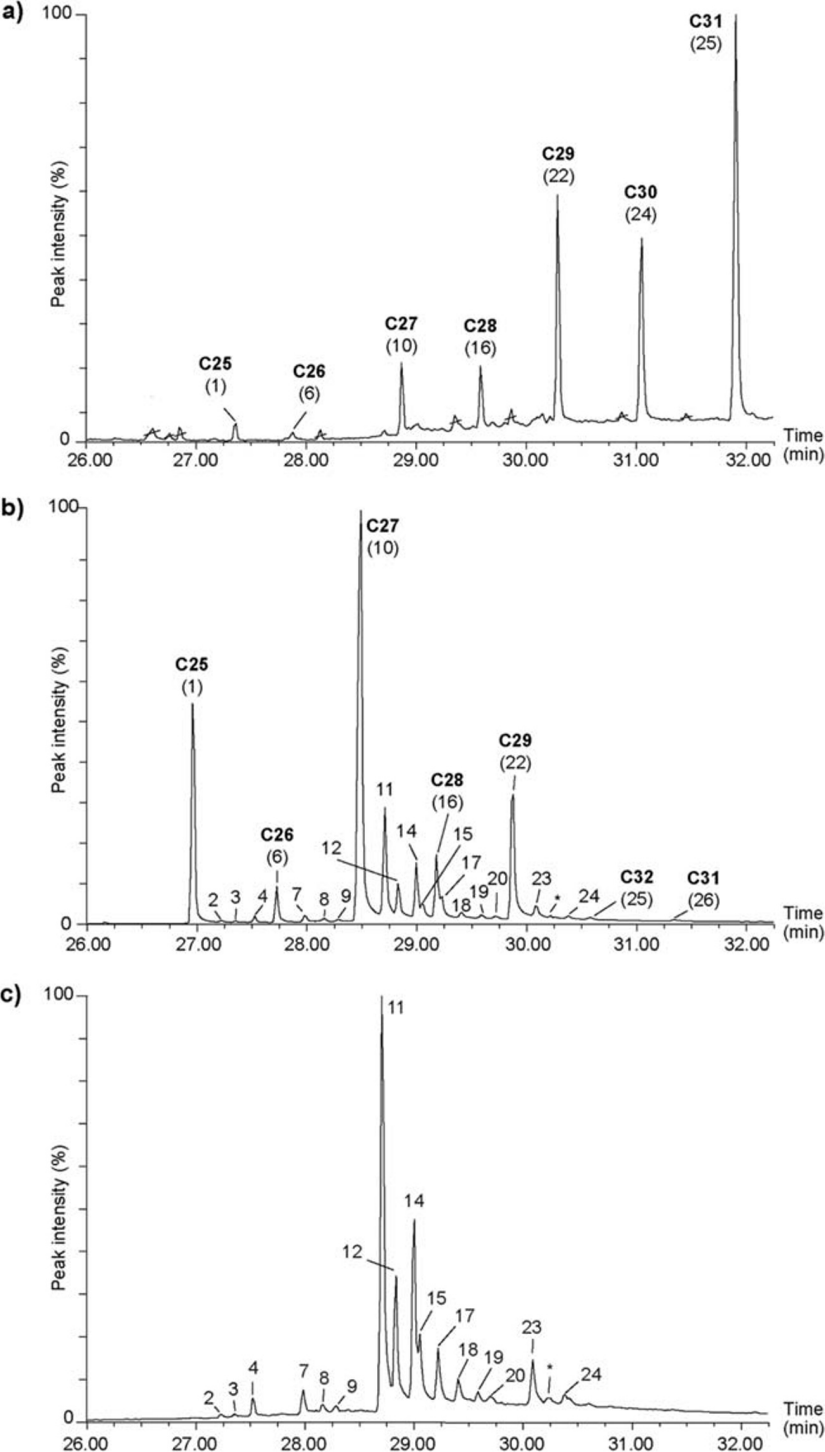
Partial total ion chromatograms (TIC) of **a**) an un-fractionated *O. surinamensis* crude larval extract, **b**) an un-fractionated *T. confusum* crude larval extract (representative for all three *Tribolium* species as the CHC profiles were similar in their composition with respect to the presence of *n*-alkanes and methyl alkanes) and **c**) the fraction of methyl alkanes of fractionated *T. confusum* crude larval extract. Numbers above peaks represent the CHCs listed in Table 2 and Table S5. The *n*-alkanes (*n*-C25 – *n*-C31) detected in the extracts of *T. confusum* and *O. surinamensis* are highlighted in bold. Crossed-out compounds are contaminations

Our GC-MS analysis revealed that crude larval extracts of *O. surinamensis* and *Tribolium* spp. exclusively consist of saturated cuticular hydrocarbons (CHCs) with chain lengths from *n*-C25 to *n*-C36. In total, we identified 31 compounds, including 12 *n*-alkanes, 14 monomethyl alkanes and five dimethyl alkanes (Table 2; Fig. 1a, b). The CHC profiles showed quantitative and qualitative differences between beetle species, but a common pattern within the genus *Tribolium* was observed (Table 2).

**Table 2:** Cuticular hydrocarbons identified from crude larval extracts of *Tribolium confusum*, *T. castaneum*, *T. destructor* and *Oryzaephilus surinamensis*

				*T. confusum*^e^		*T. castaneum*^e^		*T. destructor*^e^		*O. surinamensis*^e^	
No.^a^	Compound^b^	RI_cal_^c^	RI_lit_^d^	Mean ± SE (ng)	(%)	Mean ± SE (ng)	(%)	Mean ± SE (ng)	(%)	Mean ± SE (ng)	(%)
1	*n*-C25	2498	2500	3.35 ± 0.39	4.40	2.15 ± 0.20	3.11	16.76 ± 2.11	24.04	0.07 ± 0.02	1.90
2	11-/13-MeC25	2533	2534			0.05 ± 0.00	0.07	0.08 ± 0.01	0.11		
3	5-MeC25	2548	2550					0.36 ± 0.04	0.55		
4	3-MeC25	2572	2571	0.04 ± 0.00	0.05	0.27 ± 0.03	0.38	0.43 ± 0.05	0.64		
5	5,11-DiMeC25	2581	2577					0.32 ± 0.03	0.50		
6	*n*-C26	2597	2600	0.67 ± 0.05	0.87	0.53 ± 0.03	0.78	1.89 ± 0.15	2.83	0.02 ± 0.00	0.65
7	10-/11-/12-MeC26	2634	2632	0.09 ± 0.01	0.11	0.10 ± 0.01	0.15				
	12-/13-MeC26							0.32 ± 0.03	0.48		
8	4-MeC26	2656	2656	0.03 ± 0.00	0.03			0.13 ± 0.01	0.20		
9	3-MeC26	2674	2673			0.13 ± 0.02	0.18	0.25 ± 0.03	0.37		
10	*n*-C27	2700	2700	40.37 ± 4.36	49.55	19.13 ± 0.95	28.00	25.06 ± 1.53	38.63	0.16 ± 0.02	4.51
11	11-/13-MeC27	2731	2731	2.28 ± 0.18	2.98	4.32 ± 0.29	6.29	4.75 ± 0.33	7.30		
12	5-MeC27	2748	2750	0.58 ± 0.06	0.72			0.80 ± 0.06	1.22		
13	11,15-DiMeC27	2758	2762			0.35 ± 0.03	0.51				
14	3-MeC27	2771	2773	1.16 ± 0.13	1.39	1.04 ± 0.07	1.52	1.52 ± 0.11	2.28		
15	5, X-DiMeC27	2778	2781	0.35 ± 0.04	0.43			1.95 ± 0.13	3.01		
16	*n*-C28	2797	2800	5.84 ± 0.68	7.04	3.24 ± 0.19	4.71	1.78 ± 0.11	2.87	0.13 ± 0.01	3.46
17	3,X-DiMeC28	2802	2807					2.15 ± 0.12	3.37		
18	12-/13-/14-MeC28	2830	2831	0.21 ± 0.02	0.26	0.20 ± 0.02	0.29	0.33 ± 0.02	0.51		
19	4-MeC28	2855	2856	0.09 ± 0.01	0.11	0.03 ± 0.00	0.05				
20	3-MeC28	2874	2872			0.00 ± 0.00	0.00				
21	4,14-DiMeC28	2886	2893					0.10 ± 0.01	0.16		
22	*n*-C29	2899	2904	19.92 ± 2.96	22.71	30.36 ± 1.67	44.29	1.66 ± 0.08	2.73	0.45 ± 0.03	12.50
23	11-/13-MeC29	2928	2931	0.33 ± 0.03	0.41						
	11-/13-/15-MeC29					0.35 ± 0.03	0.51				
	11-/15-MeC29							0.18 ± 0.01	0.28		
24	3-MeC29	2970	2978	0.22 ± 0.03	0.26	0.33 ± 0.02	0.47				
25	*n*-C30	2995	3000	1.33 ± 0.13	2.01	1.51 ± 0.11	2.22	1.22 ± 0.11	2.05	0.48 ± 0.05	12.55
26	*n*-C31	3094	3100	2.02 ± 0.17	2.85	2.26 ± 0.19	3.29	1.50 ± 0.14	2.52	0.97 ± 0.08	26.04
27	*n*-C32	3194	3200	1.19 ± 0.15	1.85	1.23 ± 0.13	1.80	1.23 ± 0.12	2.08	0.60 ± 0.08	14.60
28	*n*-C33	3295	3300	1.46 ± 0.16	1.96	0.97 ± 0.10	1.40	0.76 ± 0.08	1.27	0.49 ± 0.08	11.66
29	*n*-C34	3395	3400							0.29 ± 0.06	6.52
30	*n*-C35	3495	3500							0.18 ± 0.04	3.95
31	*n*-C36	3595	3600							0.08 ± 0.02	1.66
	Total			81.63 ± 8.53	99.99	68.52 ± 3.57	100.01	65.59 ± 3.60	100.00	3.93 ± 0.42	100.00

Mean amounts (ng ± SE larva^− 1^) and relative quantities (% in 1 larval equivalent, LE) are given

^a^Peak numbers refer to Fig. 1a, b

^b^For the identification procedure see experimental part

^c^RI_cal_ = Retention index calculated on a HP-5MS capillary column (30 m x 0.25 mm x 0.25 µm)

^d^RI_lit_ = Retention index as reported for compounds analyzed on HP-5MS or similar columns in the database (http://www.pherobase.com/) and by Fürstenau and Hilker (2017) or others (peak 5 in Hebanowska et al. (1989, 1990) and Svensson et al. (2014); peak 9 in Gerhardt et al. (2016); peak 13 in Spiewok et al. (2006); peak No. 21 in Geiselhardt et al. (2009)). The provided literature RI values of dimethyl alkanes (entries 15 and 17) refer to unambiguously identified compounds described as 5,13-diMeC27 and 3,13-diMeC27

^e^For the preparation of extracts see experimental part

In *Tribolium* spp., a series of *n*-alkanes with chain lengths from *n*-C25 to *n*-C33 represented the dominating group of substance in quantities of 93% (*T. confusum*), 90% (*T. castaneum*), and 79% (*T. destructor*). Most prominent components were *n*-C27 (*T. confusum* and *T. destructor*, entry 10) and *n*-C29 (*T. castaneum*, entry 22). *n*-Alkanes with more than 30 carbon atoms (entries 25, 26, 27) accounted for less than 10% of all CHC profiles analyzed from larvae of this genus. In addition to the fraction of *n*-alkanes, various monomethyl- and dimethyl alkanes were identified in larval extracts of the *Tribolium* spp. Based on the position of the methyl branch, monomethyl alkanes were either internally branched (branching at positions 10, 11, 12, 13, 14, 15 – since positional isomers could not be separated, co-occurrence is indicated by a slash) or externally branched (branching at position 3, 4 or 5), but both groups were present in all *Tribolium* extracts. Dimethyl alkanes were most abundant in the extracts of *T. destructor* (entries 5, 15, 17, 21), while only one dimethyl alkane was present in *T. confusu*m (entry 15) and *T. castaneum* (entry 13). The mass spectra of two dimethyl alkanes (5,X-DiMeC27 and 3,X-DiMeC28; entries 15 and 17) did not allow a clear determination of the position of the second methyl group. Therefore, the unknown position was labelled by ‘X’ (Table 2). Overall, seven methyl alkanes were found in all extracts of *Tribolium* spp. (3-MeC25, 11-/13-MeC27, 3-MeC27 and 12-/13-/14-MeC28; entries 4, 11, 14 and 18) (Table 2). Among these compounds, 11-MeC27 and 13-MeC27 were most abundant (*T. confusum* = 3%, *T. castaneum* = 6%, *T. destructor* = 7%).

In contrast to the CHC profiles of *Tribolium* spp., the profile of *O. surinamensis* was less diverse and contained only linear alkanes, but no methyl alkanes. In total, the assigned peaks represented *n*-alkanes with chain lengths from *n*-C25 to *n*-C36 (Table 2; Fig. 1a). The most abundant component was *n*-C31 (entry 26), and compounds with more than 29 carbon atoms contributed to 77% of the total peak area (Table 2).

**Fig. 2:**
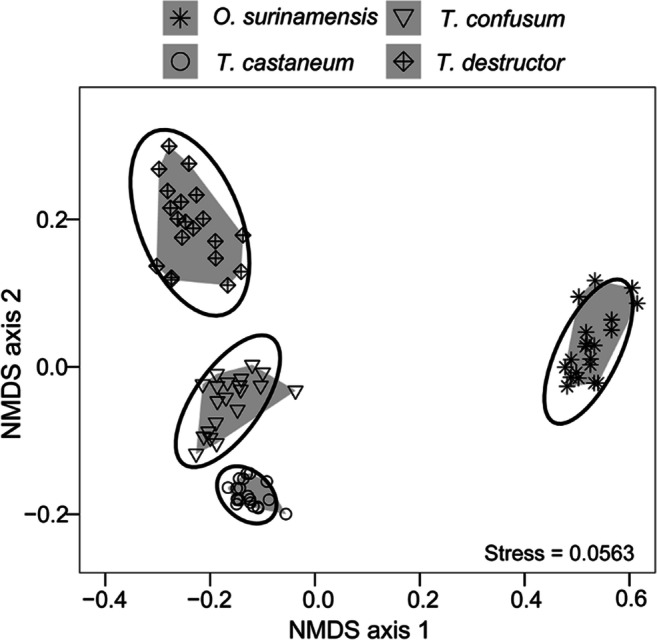
Comparison of larval CHC profiles of *T. castaneum*, *T. confusum*, *T. destructor* and *O. surinamensis* (*N* = 20 per species) visualized by a non-metric multi-dimensional scaling (NMDS) calculated for two dimensions. The ellipses show the 95%-confidence areas around each centroid for each species

Comparison of the CHC profiles of *Tribolium* spp. larvae among each other and with those of *O. surinamensis* larvae by an ANOSIM showed a significant dissimilarity among the beetle species (*R =* 0.9988, *P* < 0.001), which were clearly separated in the NDMS plot (Fig. 2). *Tribolium* spp., however, clustered closer together, indicating that the composition of their CHC profiles was more similar compared to *O. surinamensis*. This was confirmed by the overall average dissimilarity index calculated in SIMPER (Table S1); this index was smaller when comparing the *Tribolium* spp. among each other (*T. castaneum* vs. *T. confusum* vs. *T. destructor* = 38.96) than in the pairwise comparison between each *Tribolium* spp. and *O. surinamensis* (*T. castaneum* vs. *O. surinamensis* = 68.75, *T. confusum* vs. *O. surinamensis* = 68.58, *T. destructor* vs. *O. surinamensis* = 79.79, Table S2, S3, S4). In addition, the SIMPER analysis revealed that all compounds, which accounted most for the dissimilarity between *Tribolium* spp. as well as between each *Tribolium* spp. and *O. surinamensis*, were *n*-alkanes (> 10%). When considering the within-genus comparison of *Tribolium* spp., *n-*C25 (entry 1), *n-*C27 (entry 10), and *n*-C29 (entry 22) contributed together by 73% to the observed dissimilarity (Table S1).

Fractionation of *T. confusum* larval extracts resulted in a clear separation of linear *n*-alkanes on the one hand and monomethyl and dimethyl alkanes on the other (Fig. 1b, c). All methyl alkanes, which had been previously detected in the un-fractionated crude larval extracts, were recovered after fractionation, but in smaller amounts (Table S5). Furthermore, five compounds were detected in the fraction of methyl alkanes (entries 2, 3, 17, 20 and *), which were not found by our GC-MS analysis of whole *T. confusum* crude larval extracts (Table 2).

### Host Finding and Recognition

To test whether the parasitoids’ host recognition behavior is mediated by compounds present in the larval CHC profile of all possible host species, we conducted several contact bioassays (Table 1).

**Fig. 3:**
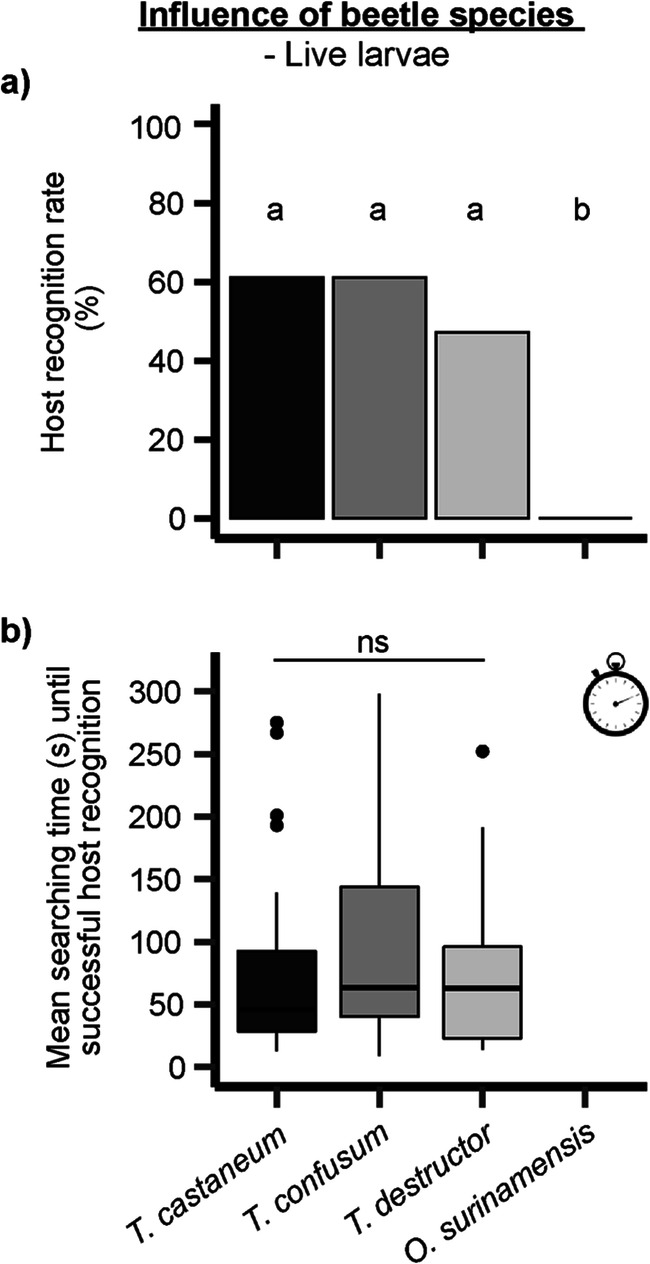
Behavioral responses of female *H. sylvanidis* in contact bioassays to live larvae of potential host species (*T. castaneum*, *T. confusum*, *T. destructor* and *O. surinamensis*; *N* = 36 per species, max. observation time = 300 s). **a**) Host recognition rate (100% ≙ 36 successful host recognition events per beetle species) was analyzed by the test for equality of proportions followed by pairwise comparison of proportions with *Bonferroni-Holm* correction. Different letters indicate significant differences at *P* < 0.05. **b)** Mean searching time until successful host recognition was analyzed for *Tribolium* spp. and not for *O. surinamensis* as larvae of the latter species were rejected as potential hosts for the parasitoid. Statistical analysis was performed by *Kruskal-Wallis* test (ns = not significant, *P* > 0.05)

When live larvae of different beetle species were offered, *H. sylvanidis* recognized more than 60% of the *T. castaneum* and *T. confusum* larvae as hosts (Fig. 3a). Although the host recognition rate of *T. destructor* larvae was lower (47%), it did not differ significantly among *Tribolium* spp., but was significantly higher compared to the rate of *O. surinamensis* larvae (*χ²* = 37.173, df = 3, *P* < 0.001). In contrast, the host recogntion rate decreased in all *Tribolium* spp. when dead larvae were tested (Figure S2a). Nevertheless, the proportion of successfuly recognized host larvae was significantly higher in *Tribolium* spp. than in *O. surinamensis* (*χ²* = 30.424, df = 3, *P* < 0.001). Live and dead *O. surinamensis* larvae were not recognized as hosts (Fig. 3a, S2a).

*Holepyris sylvanidis* tended to locate live larvae of *T. castaneum* (Figs. 3b and 80.6 ± 17.3 s) and *T. destructor* (79.4 ± 16.8 s) faster than those of *T. confusum* (102.8 ± 18.4 s). However, the mean searching times until successful host recognition were not significantly different (*χ²* = 1.42, df = 2, *P* = 0.49). When dead larvae were offered, the mean searching time increased in all *Tribolium* spp. (Figure S2b, *T. castaneum* = 115.3 ± 21.3 s, *T. confusum* = 134.1 ± 21.1 s, *T. destructor* = 109.3 ± 20.7 s), but did not differ significantly among them (*χ²* = 0.66, df = 2, *P* = 0.72).

**Fig. 4:**
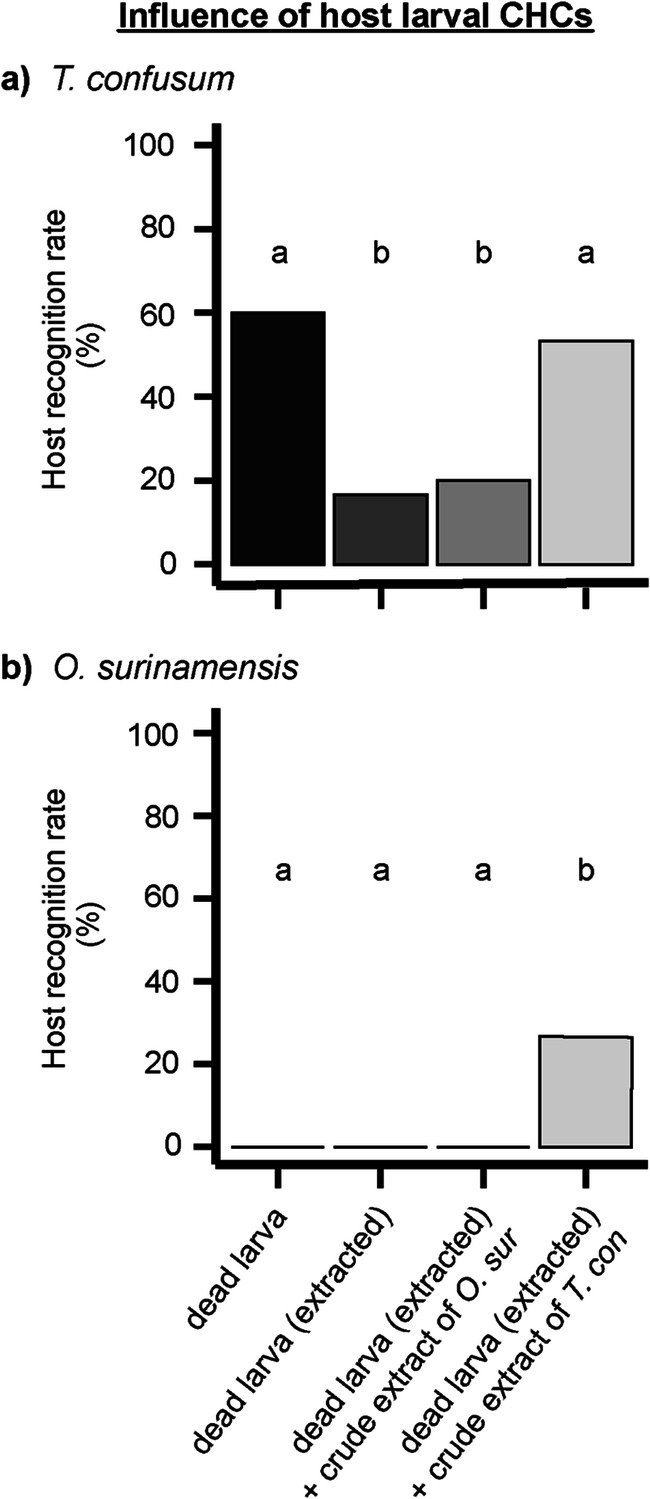
Host recognition rate in contact bioassays of female *H. sylvanidis* exposed to freshly killed larvae of **a**) *T. confusum* (*T. con*) and **b**) *O. surinamensis* (*O. sur*) treated as follows (*N* = 30 per species and stimulus, 100% ≙ 30 successful host recognition events per stimulus, max. observation time = 300 s): (i) untreated, (ii) extracted with *n*-hexane, (iii) extracted with *n*-hexane and treated with crude larval extract of *O. surinamensis* (½ LE), (iv) extracted with *n*-hexane and treated with crude larval extract of *T. confusum* (½ LE). Host recognition rate was analyzed by the test for equality of proportion (*T. confusum*) or *Fisher’s* exact test (*O. surinamensis*) followed by pairwise comparison of proportions with *Bonferroni-Holm* correction. Different letters indicate significant differences at *P* < 0.05

Based on these results, we selected *T. confusum* as representative host species for *Tribolium* spp. and *O. surinamensis* as non-host species for subsequent contact bioassays to investigate the influence of different larval CHC profiles on the host recognition behavior of *H. sylvanidis* (Table 1). Parasitoid females responded significantly different to the four treatments of *T. confusum* larvae (Fig. 4a, *χ*² = 19.164, df = 3, *P* < 0.001; treatments #5–8 in Table 1). While 60% of untreated larvae were successfully recognized as hosts, larvae became significantly less attractive for the parasitoid, when CHCs had been removed by solvent extraction (17%). Applying a CHC extract of *O. surinamensis* larvae onto previously extracted *T. confusum* larvae, led to a slightly increased amount of successfully recognized host larvae (20%). However, this number of successfully recognized larvae did not significantly differ from the one recorded in the previous treatment with extracted larvae. In contrast, the attractiveness of extracted *T. confusum* larvae was restored after applying a CHC extract of *T. confusum* larvae (53%, Fig. 4a). Furthermore, *O. surinamensis* larvae became only attractive to parasitoid females after extracting their CHCs and applying a larval extract of *T. confusum* (Fig. 4b, *P* < 0.001; treatments #9–12 in Table 1). No statistical differences in the mean searching time of *H. sylvanidis* were found between the different combinations of treatments and larvae (Figure S3, *χ²* = 4.836, df = 4, *P* = 0.305).

**Fig. 5:**
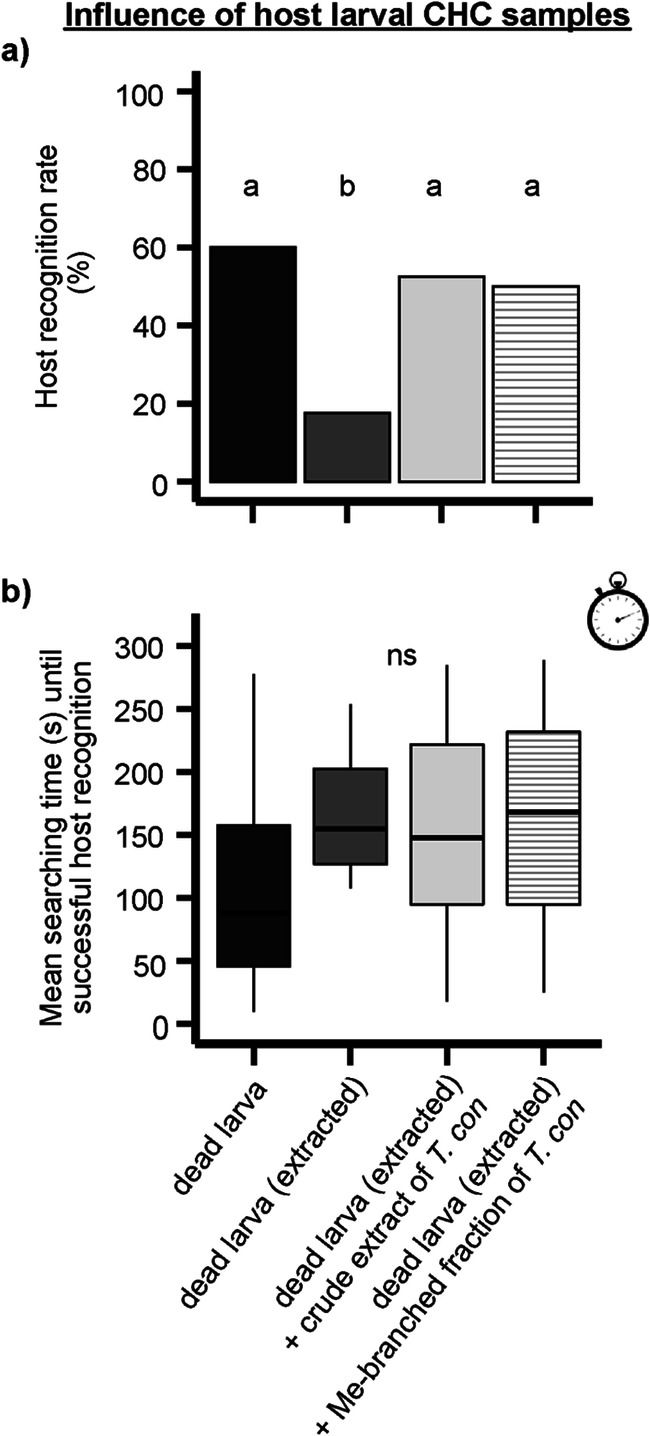
Behavioral responses of female *H. sylvanidis* to freshly killed *T. confusum* larvae treated as follows (*N* = 40 per stimulus): (i) untreated, (ii) extracted with *n*-hexane, (iii) extracted with *n*-hexane and treated with an un-fractionated larval extract of *T. confusum* (½ LE), (iv) extracted with *n*-hexane and treated with the fraction of methyl alkanes from fractionated *T. confusum* crude larval extract (½ LE). **a)** Host recognition rate (100% ≙ 40 successful host recognition events per stimulus) was analyzed by the test for equality of proportions followed by pairwise comparison of proportions with *Bonferroni-Holm* correction. Different letters indicate significant differences at *P* < 0.05. **b)** Mean searching time until successful host recognition was analyzed by *Kruskal-Wallis* test (ns = not significant, *P* > 0.05)

To elucidate which substance group within the CHC profile of *T. confusum* might be responsible for host recognition of *H. sylvanidis*, different CHC fractions were tested in contact bioassays. Applying the fraction of methyl alkanes onto previously extracted larvae was sufficient to induce host recognition behavior in parasitoid females (Fig. 5a; treatments #13–16 in Table 1). The host recogntion rate (50%) was not significantly different from that of untreated larvae (60%) as well as of larvae, which had been extracted and treated with whole larval extracts (un-fractionated, 53%). In these three treatments significantly more larvae were successfully recognized as hosts compared to the treatment in which previously extracted larvae were offered (18%; *χ²* = 17.172, df = 3, *P* < 0.001). Although *H. sylvanidis* located and recognized untreated *T. confusum* larvae slightly faster than the treated ones, the mean searching time did not differ among treatments #13–16 (Table 1; *χ²* = 7.196, df = 3, *P* = 0.07; Fig. 5b).

### Host Acceptance

Neither live nor freshly killed *O. surinamensis* larvae had been recognized as hosts in contact bioassays, but we included larvae of this beetle species for control purposes (e.g. to test the possibility that the lack of host recognition response might be due to a short test duration (*t* = 300 s)).

After exposure of larvae to the parasitoid for 24 h, more than 50% of the 40 offered larvae of each *Tribolium* species were parasitized (*T. castaneum* = 29, *T. confusum* = 27, *T. destructor* = 23; Table S6). The parasitoid successfully completed its development from egg to adult on more than 89% of all parasitized host larvae within four weeks (*T. castaneum* = 26, *T. confusum* = 25, *T. destructor* = 22; Table S6). Most parasitoid adults emerged from parasitized *T. castaneum* larvae (hatching rate = 65%), but no significant differences among *Tribolium* spp. were observed (*T. confusum* = 63%, *T. destructor* = 55%; Figure S4). No parasitoid eggs were laid on *O. surinamensis* larvae within a 24 h exposure period and thus, host acceptance of this species significantly differed from those of *Tribolium* spp. (*χ²* = 45.624, df = 3, *P* < 0.001).

## Discussion

Our study shows that a larval ectoparasitoid, *H. sylvanidis*, which is supposed to be polyphagous on beetle larvae infesting stored products, selects its hosts by responding to methyl alkanes of the host cuticle. While live and dead 4th instars of the tested *Tribolium* spp. were successfully recognized as hosts by parasitoid females, larvae of *O. surinamensis* were not, although it had been described as host species in the past (Amante et al. 2017b; Evans 1969; Hagstrum and Subramanyam 2009). Chemical analysis of crude larval extracts revealed that the CHC profiles of *Tribolium* spp. and those of *O. surinamensis* varied qualitatively and quantitatively. The cuticular extracts of the four investigated beetle species contained *n*-alkanes. The major difference between the CHC patterns was the presence of methyl alkanes, which represented a minor fraction on the cuticle of *Tribolium* spp., but were absent in *O. surinamensis.* A series of contact bioassays with dead and live larvae of the different beetle species showed that the parasitoid recognizes and accepts a potential host based on the presence of methyl alkanes on the cuticle of an offered larvae. Moreover, a similar response in *H. sylvanidis* was provoked by treating dead, extracted *T. confusum* larvae solely with a fraction of methyl alkanes isolated from *T. confusum* larvae. Therefore, we suggest that host recognition behavior of *H. sylvanidis* is most likely mediated by methyl alkanes from the cuticle of *Tribolium* spp. larvae, whereas the presence of *n-*alkanes alone is not sufficient to elicit host recognition in the parasitoid.

To the best of our knowledge, our results show for the first time that methyl alkanes are essential to elicit host recognition behavior of a parasitoid species. However, methyl alkanes are well known to elicit behavioral responses in sexual communicaton of parasitoid species belonging to the Encrytidae (Ablard et al. 2012) and Pteromalidae (Kühbandner et al. 2012b; Ruther et al. 2011; Steiner et al. 2005, 2007) here the methyl alkanes serve as contact sex pheromones. A recent study by Würf et al. (2020) of a pteromalid species suggests that cuticular methyl alkanes may also act synergistically with *n*-alkanes as contact sex pheromone. For a broad range of non-parasitic insect taxa, methyl alkanes have also been described as contact pheromones, among them ants, beetles, thrips, and psyllids (De Narbonne et al. 2016; Ginzel et al. 2003a; Guédot et al. 2009; Holman et al. 2010; Lacey et al. 2008; Olaniran et al. 2013; Silk et al. 2011; Spikes et al. 2010; Sugeno et al. 2006). For instance, it has been shown that ants use methyl alkanes to discriminate between a mutualistic partner, aphid species, and a non-partner (Sakata et al. 2017).

To study how different larval CHC profiles and isolated methyl alkanes influence host recognition behavior of *H. sylvanidis*, we used solvent extracted *T. confusum* larvae as dummy larvae as (negative) controls. Some of these larvae were still successfully recognized as hosts by the parasitoid in spite of having been extracted leaving no CHCs. This result suggests that not the entire CHC blend was extracted and small amounts of some cuticular lipids may have been left on the larval cuticle. Hence, we cannot fully exclude that some remaining CHCs (e.g. *n*-alkanes) and/or compounds other than CHCs might also affect the behavior of the parasitoid.

However, we observed that parasitoid females responded to solvent-extracted *T. confusum* larvae treated with isolated methyl alkanes similarly as to dead non-extracted *T. confusum* larvae with a complete blend of CHCs.

These results demonstrate the relevance of methyl alkanes for host recognition behavior of *H. sylvanidis.* So far, we cannot pinpoint yet, which methyl alkanes are most important. Neither do we know whether compounds with higher boiling points than those detected here are relevant for the parasitoids’ host foraging behavior. The CHC profiles of the closely related *Tribolium* spp. were slightly different. Nevertheless, larvae of each of these beetle species were successfully recognized and accepted as hosts for oviposition by *H. sylvanidis*, and the parasitoid progenies emerged successfully from most of the parasitized host larvae. These findings suggest that host foraging *H. sylvanidis* females rely on those methyl-branched host CHCs, which the analyzed *Tribolium* spp. have in common.

We detected 2 externally methyl-branched alkanes (3-MeC25 and 3-MeC27) and 7 internally methyl-branched alkanes (11-/13-MeC25, 11-/13-MeC27 and 12-/13-/14-MeC28), which were present in all *Tribolium*-CHC profiles; among these compounds, 11-/13-MeC27 and 3-MeC27 belonged to the most abundant methyl alkanes in the *Tribolium*-CHC profiles. These common methyl alkanes might be key components eliciting host recognition behavior in *H. sylvanidis* (entries 2, 4, 11, 14 and 18 in Table 2 and Table S5). This is supported by other studies, which also found behavioral functions for some of these compounds. In particular, 11-/13-MeC27 and 3-MeC27 are known to act as chemical cues affecting various behaviors in different insects, for example mate finding, (nest) mate and kin recognition or brood discrimination (Guédot et al. 2009; Kühbandner et al. 2012b; Salazar et al. 2015; Silk et al. 2011; Sugeno et al. 2006). Both compounds were also detected in traces laid by *T. confusum* larvae, which elicited trail-following behavior in *H. sylvanids* females (Fürstenau and Hilker 2017). Further studies must be undertaken to investigate, whether single key components are sufficient alone or in combination to elicit host recogntion behavior of *H. sylvanidis*. It will be necessary to carry out similar bioassays with synthetic methyl alkanes to determine those that are essential for host recognition in *H. sylvanidis*.

Moreover, the chirality of methyl CHCs should be considered since previous studies have shown that enantiomers of a certain compound and its racemate may evoke different behavioral responses in parasitoids (Silk et al. 2011; Würf et al. 2020). For instance, males of the egg parasitoid *Ooencyrtus kuvanae* were highly attracted to a blend of 5*S*-MeC27 and (5*R*,17*S*)-DiMeC27, whereas they were repelled by a blend of 5*R*-MeC27 and (5*R*,17*R*)-DiMeC27 (Ablard et al. 2012).

Larvae of *O. surinamensis* have previously been described as potential host of *H. sylvanidis* since both the parasitoid and beetle species have been found at the same storage facilities (e.g. Eliopoulos et al. 2002). However, the chemically mediated parasitoid-host interaction had not been investigated prior to our study, although the CHC profiles of *O. surinamensis* larvae and adults have been examined (Howard et al. 1995). In this study, a different and more diverse composition of CHCs was described compared to our analyses. Besides several *n*-alkanes with a chain length from *n*-C21 to *n*-C35, which represented the predominant group of compounds in the CHC profiles, the authors also identified few alkenes and even some methyl alkanes, which we did not find in our investigation. Possible explanations for the differences in the CHC composition of *O. surinamensis* between the study by Howard et al. (1995) and ours may be the origin of test individuals (different lab strains), different rearing conditions (feeding substrate) and the developmental stage. Howard et al. (1995) did not discriminate between larvae and adults in their analyses of the *O. surinamensis* CHC profiles. Tested insects had been kept on a mix of whole wheat flour enriched with 5% brewer’s yeast and rolled oats. In contrast, we used only beetle larvae for our analysis and provided them with coarsely ground wheat grist.

From previous studies it is known that insect CHC profiles may vary depending on strain, age, and diet (see references in Otte et al. 2018; Ngumbi et al. 2020). In particular, changes in the insects’ diet may lead to shifts in the composition of CHC profiles since the uptake of amino acids and fatty acids is essential for their biosynthesis, especially that of methyl alkanes (Blomquist and Bagnères 2010; Otte et al. 2014). These diet-induced shifts of CHC compositions can proceed very fast within a short period of one generation or two weeks, thus altering interactions between insects (Geiselhardt et al. 2012; Kühbandner et al. 2012a). Nevertheless, we observed in our contact bioassays that all attempts of *H. sylvanidis* to antennate *O. surinamensis* were aggressively repulsed by the larvae. To exclude this defense behavior, dead larvae were offered as potential hosts to parasitoid females, but they were still not accepted as hosts, most probably due to the lack of methyl alkanes on their cuticle. Therefore, we consider it rather unlikely that *O. surinamensis* is a suitable host for *H. sylvanidis*.

In summary, the present study demonstrates that methyl alkanes present on the cuticle of *Tribolium* larvae mediate host recognition in *H. sylvanidis* and enable parasitoid females to differentiate among host and non-host species. Furthermore, the presence of several identical methyl alkanes in all CHC profiles of *Tribolium* larvae indicates that parasitoid females identify suitable hosts by using these compounds as key substances. Since *Tribolium* spp. are worldwide pests of various stored products, it is very likely that diet-induced shifts in the composition of CHCs can occur. Future systematic studies need to address, how changes in the diet can affect the presence of methyl alkanes on the cuticle of host larva. If a diet shift results in significant changes in the pattern of methyl alkanes of host larvae, further studies should elucidate, how such changes affect host recognition by *H. sylvanidis*.

## Supplementary Information

ESM 1 (DOCX 968 KB)
