# Supplementary material for: The Importance of Methyl-Branched Cuticular Hydrocarbons for Successful Host Recognition by the Larval Ectoparasitoid Holepyris sylvanidis
Source: J Chem Ecol. 2020 Oct 29;46(11):1032–46. doi: 10.1007/s10886-020-01227-w (PMC7677283; doi:10.1007/s10886-020-01227-w)
Supplement: Supplementary file 1 — (DOCX 968 KB) [file 10886_2020_1227_MOESM1_ESM.docx]

**ELECTRONIC SUPPLEMENTARY MATERIAL**

**The importance of methyl-branched cuticular hydrocarbons for successful host recognition by the larval ectoparasitoid *Holepyris sylvanidis***

Journal Of Chemical Ecology

Sarah Awater-Salendo^1,2^, Hartwig Schulz^1^, Monika Hilker^2^, Benjamin Fürstenau^1*^

*^1^ Julius Kühn Institute, Federal Research Centre for Cultivated Plants, Institute for Ecological Chemistry, Plant Analysis and Stored Product Protection, Königin-Luise-Str.19, 14195 Berlin, Germany*

*² Dahlem Centre of Plant Science, Institute of Biology, Applied Zoology/Animal Ecology, Freie Universität Berlin, Haderslebener Str.9, 12163 Berlin, Germany*

***** Author for correspondence (e-mail: [*benjamin.fuerstenau@julius-kuehn.de*](mailto:Benjamin.fuerstenau@julius-kuehn.de), Tel.: +49 30 8304 2080)

**Tables**

**Table S1** Results of the SIMPER analysis comparing larval cuticular hydrocarbon profiles of three Tribolium species (T. castaneum (T. cas), T. confusum (T. con) and T. destructor (T. des); overall average dissimilarity index = 38.96)

| **Compound** | **Average~~.~~ dissimilarity** | **Contribution (%)** | **Cumulative (%)** | ***T. cas*** | ***T. con*** | ***T. des*** |
| --- | --- | --- | --- | --- | --- | --- |
| *n*-C29 | 13.85 | 35.55 | 35.55 | 44.30 | 22.70 | 2.72 |
| *n*-C27 | 7.45 | 19.13 | 54.68 | 28.00 | 49.50 | 38.60 |
| *n*-C25 | 7.09 | 18.21 | 72.88 | 3.11 | 4.40 | 24.00 |
| 11-/13-MeC27 | 1.58 | 4.05 | 76.93 | 6.29 | 2.97 | 7.29 |
| *n*-C28 | 1.40 | 3.59 | 80.52 | 4.71 | 7.03 | 2.86 |
| 3,X-DiMeC28 | 1.12 | 2.88 | 83.40 | 0.00^†^ | 0.00^†^ | 3.37 |
| 5,X-DiMeC27 | 1.00 | 2.21 | 85.97 | 0.00^†^ | 0.43 | 3.01 |
| *n*-C31 | 0.77 | 1.99 | 87.96 | 3.29 | 2.85 | 2.52 |
| *n*-C26 | 0.70 | 1.79 | 89.75 | 0.78 | 0.87 | 2.83 |
| *n*-C32 | 0.63 | 1.62 | 91.37 | 1.80 | 1.85 | 2.08 |
| *n*-C30 | 0.60 | 1.54 | 92.91 | 2.22 | 2.01 | 2.05 |
| *n*-C33 | 0.47 | 1.21 | 94.13 | 1.40 | 1.96 | 1.27 |
| 5-MeC27 | 0.41 | 1.05 | 95.17 | 0.00^†^ | 0.72 | 1.22 |
| 3-MeC27 | 0.33 | 0.84 | 96.02 | 1.52 | 1.39 | 2.27 |
| 3-MeC25 | 0.21 | 0.55 | 96.57 | 0.00^†^ | 0.06 | 0.64 |
| 5-MeC25 | 0.19 | 0.50 | 97.06 | 0.38 | 0.00^†^ | 0.55 |
| 11,15-DiMeC27 | 0.17 | 0.44 | 97.50 | 0.51 | 0.00^†^ | 0.00^†^ |
| 5,11-DiMeC25 | 0.17 | 0.43 | 97.93 | 0.00^†^ | 0.00^†^ | 0.50 |
| 3-MeC29 | 0.16 | 0.41 | 98.34 | 0.47 | 0.28 | 0.00^†^ |
| 3-MeC26 | 0.13 | 0.33 | 98.67 | 0.18 | 0.00^†^ | 0.37 |
| 10-/11-/12-/13-MeC26* | 0.12 | 0.32 | 98.98 | 0.15 | 0.12 | 0.48 |
| 12-/13-/14-MeC28 | 0.09 | 0.24 | 99.23 | 0.29 | 0.26 | 0.51 |
| 11-/13-MeC29* | 0.08 | 0.21 | 99.44 | 0.51 | 0.41 | 0.29 |
| 4-MeC26 | 0.07 | 0.18 | 99.62 | 0.00^†^ | 0.04 | 0.22 |
| 4,14-DiMeC28 | 0.06 | 0.15 | 99.77 | 0.00^†^ | 0.00^†^ | 0.17 |
| 11-/13-MeC25 | 0.05 | 0.14 | 99.90 | 0.07 | 0.00^†^ | 0.16 |
| 4-MeC28 | 0.04 | 0.09 | 100.00 | 0.05 | 0.11 | 0.00^†^ |
| 3-MeC28 | 0.00 | 0.00 | 100.00 | 0.00 | 0.00^†^ | 0.00^†^ |
| *n*-C36 | 0.00 | 0.00 | 100.00 | 0.00^†^ | 0.00^†^ | 0.00^†^ |
| *n*-C35 | 0.00 | 0.00 | 100.00 | 0.00^†^ | 0.00^†^ | 0.00^†^ |
| *n*-C34 | 0.00 | 0.00 | 100.00 | 0.00^†^ | 0.00^†^ | 0.00^†^ |

* Compounds with similar RI were pooled despite different positions of the methyl group

^†^ Compound is no component in the specific cuticular hydrocarbon profile of the three beetle species. For the SIMPER analysis, the value of this compound was determined as ‘0.00’

**Table S2** Results of the SIMPER analysis comparing larval cuticular hydrocarbon profiles of T. castaneum (T. cas) and O. surinamensis (O. sur) (overall average dissimilarity index = 68.75)

| **Compound** | **Average~~.~~ dissimilarity** | **Contribution (%)** | **Cumulative (%)** | ***T. cas*** | ***O. sur*** |
| --- | --- | --- | --- | --- | --- |
| *n*-C29 | 15.89 | 23.11 | 23.11 | 44.30 | 12.50 |
| *n*-C27 | 11.75 | 17.09 | 40.20 | 28.00 | 4.51 |
| *n*-C31 | 11.38 | 16.55 | 56.75 | 3.29 | 26.00 |
| *n*-C32 | 6.40 | 9.31 | 66.06 | 1.80 | 14.60 |
| *n*-C30 | 5.17 | 7.52 | 73.57 | 2.22 | 12.60 |
| *n*-C33 | 5.13 | 7.46 | 81.03 | 1.40 | 11.70 |
| *n*-C34 | 3.26 | 4.74 | 85.77 | 0.00^†^ | 6.52 |
| 11-/13-MeC27 | 3.15 | 4.58 | 90.35 | 6.29 | 0.00^†^ |
| *n*-C35 | 1.97 | 2.87 | 93.22 | 0.00^†^ | 3.95 |
| *n*-C25 | 0.92 | 1.34 | 94.56 | 3.11 | 1.90 |
| *n*-C36 | 0.83 | 1.21 | 95.77 | 0.00^†^ | 1.67 |
| 3-MeC27 | 0.76 | 1.11 | 96.88 | 1.52 | 0.00^†^ |
| *n*-C28 | 0.72 | 1.04 | 97.92 | 4.71 | 3.46 |
| 11,15-DiMeC27 | 0.25 | 0.37 | 98.29 | 0.51 | 0.00^†^ |
| 11-/13-MeC29* | 0.25 | 0.37 | 98.66 | 0.51 | 0.00^†^ |
| 3-MeC29 | 0.24 | 0.34 | 99.00 | 0.47 | 0.00^†^ |
| 5-MeC25 | 0.19 | 0.27 | 99.27 | 0.38 | 0.00^†^ |
| 12-/13-/14-MeC28 | 0.14 | 0.21 | 99.48 | 0.29 | 0.00^†^ |
| *n*-C26 | 0.13 | 0.19 | 99.67 | 0.78 | 0.65 |
| 3-MeC26 | 0.09 | 0.13 | 99.80 | 0.18 | 0.00^†^ |
| 10-/11-/12-/13-MeC26* | 0.07 | 0.11 | 99.91 | 0.15 | 0.00^†^ |
| 11-/13-MeC25 | 0.03 | 0.05 | 99.96 | 0.07 | 0.00^†^ |
| 4-MeC28 | 0.02 | 0.04 | 100.00 | 0.05 | 0.00^†^ |
| 3-MeC28 | 0.00 | 0.00 | 100.00 | 0.00 | 0.00^†^ |
| 5,X-DiMeC27 | 0.00 | 0.00 | 100.00 | 0.00^†^ | 0.00^†^ |
| 5-MeC27 | 0.00 | 0.00 | 100.00 | 0.00^†^ | 0.00^†^ |
| 4,14-DiMeC28 | 0.00 | 0.00 | 100.00 | 0.00^†^ | 0.00^†^ |
| 5,11-DiMeC25 | 0.00 | 0.00 | 100.00 | 0.00^†^ | 0.00^†^ |
| 3,X-DiMeC28 | 0.00 | 0.00 | 100.00 | 0.00^†^ | 0.00^†^ |
| 4-MeC26 | 0.00 | 0.00 | 100.00 | 0.00^†^ | 0.00^†^ |
| 3-MeC25 | 0.00 | 0.00 | 100.00 | 0.00^†^ | 0.00^†^ |

* Compounds with similar RI were pooled despite different positions of the methyl group

^†^ Compound is no component within the specific cuticular hydrocarbon profile of the two beetle species. For the SIMPER analysis, the value of this compound was determined as ‘0.00’

**Table S3** Results of the SIMPER analysis comparing larval cuticular hydrocarbon profiles of T. confusum (T. con) and O. surinamensis (O. sur) (overall average dissimilarity index = 68.58)

| **Compound** | **Average~~.~~ dissimilarity** | **Contribution (%)** | **Cumulative (%)** | ***T. con*** | ***O. sur*** |
| --- | --- | --- | --- | --- | --- |
| *n-*C27 | 22.51 | 32.82 | 32.82 | 49.50 | 4.51 |
| *n-*C31 | 11.59 | 16.91 | 49.72 | 2.85 | 26.00 |
| *n-*C32 | 6.38 | 9.30 | 59.02 | 1.85 | 14.60 |
| *n-*C30 | 5.27 | 7.68 | 66.71 | 2.01 | 12.60 |
| *n-*C29 | 5.09 | 7.43 | 74.13 | 22.70 | 12.50 |
| *n-*C33 | 4.85 | 7.07 | 81.20 | 1.96 | 11.70 |
| *n-*C34 | 3.26 | 4.75 | 85.96 | 0.00^†^ | 6.52 |
| *n-*C35 | 1.97 | 2.88 | 88.84 | 0.00^†^ | 3.95 |
| *n-*C28 | 1.79 | 2.61 | 91.45 | 7.03 | 3.46 |
| 11-/13-MeC27 | 1.49 | 2.17 | 93.61 | 2.97 | 0.00^†^ |
| *n-*C25 | 1.47 | 2.14 | 95.75 | 4.40 | 1.90 |
| *n-*C36 | 0.83 | 1.21 | 96.97 | 0.00^†^ | 1.67 |
| 3-MeC27 | 0.70 | 1.01 | 97.98 | 1.39 | 0.00^†^ |
| 5-MeC27 | 0.36 | 0.53 | 98.51 | 0.72 | 0.00^†^ |
| 5,X-DiMeC27 | 0.22 | 0.32 | 98.82 | 0.43 | 0.00^†^ |
| 11-/13-MeC29* | 0.21 | 0.30 | 99.12 | 0.41 | 0.00^†^ |
| *n-C26* | 0.16 | 0.24 | 99.36 | 0.87 | 0.65 |
| 3-MeC29 | 0.14 | 0.21 | 99.57 | 0.28 | 0.00^†^ |
| 12-/13-/14-MeC28 | 0.13 | 0.19 | 99.76 | 0.26 | 0.00^†^ |
| 10-/11-/12-13-MeC26* | 0.06 | 0.08 | 99.84 | 0.12 | 0.00^†^ |
| 4-MeC28 | 0.06 | 0.08 | 99.92 | 0.11 | 0.00^†^ |
| 3-MeC25 | 0.03 | 0.05 | 99.97 | 0.06 | 0.00^†^ |
| 4-MeC26 | 0.02 | 0.03 | 100.00 | 0.04 | 0.00^†^ |
| 11,15-DiMeC27 | 0.00 | 0.00 | 100.00 | 0.00^†^ | 0.00^†^ |
| 5-MeC25 | 0.00 | 0.00 | 100.00 | 0.00^†^ | 0.00^†^ |
| 4,14-DiMeC28 | 0.00 | 0.00 | 100.00 | 0.00^†^ | 0.00^†^ |
| 3-MeC28 | 0.00 | 0.00 | 100.00 | 0.00^†^ | 0.00^†^ |
| 5,11-DiMeC25 | 0.00 | 0.00 | 100.00 | 0.00^†^ | 0.00^†^ |
| 3-MeC26 | 0.00 | 0.00 | 100.00 | 0.00^†^ | 0.00^†^ |
| 3,X-DiMeC28 | 0.00 | 0.00 | 100.00 | 0.00^†^ | 0.00^†^ |
| 11-/13-MeC25 | 0.00 | 0.00 | 100.00 | 0.00^†^ | 0.00^†^ |

* Compounds with similar RI were pooled despite different positions of the methyl group

^†^ Compound is no component within the specific cuticular hydrocarbon profile of the two beetle species. For the SIMPER analysis, the value of this compound was determined as ‘0.00’

**Table S4** Results of the SIMPER analysis comparing larval cuticular hydrocarbon profiles of T. destructor (T. des) and O. surinamensis (O. sur) (overall average dissimilarity index = 79.79)

| **Compound** | **Average~~.~~ dissimilarity** | **Contribution (%)** | **Cumulative (%)** | ***T. des*** | ***O. sur*** |
| --- | --- | --- | --- | --- | --- |
| *n*-C27 | 17.04 | 21.36 | 21.36 | 38.60 | 4.51 |
| *n*-C31 | 11.76 | 14.74 | 36.10 | 2.52 | 26.00 |
| *n*-C25 | 11.06 | 13.86 | 49.96 | 24.00 | 1.90 |
| *n*-C32 | 6.26 | 7.85 | 57.81 | 2.08 | 14.60 |
| *n*-C30 | 5.25 | 6.58 | 64.39 | 2.05 | 12.60 |
| *n*-C33 | 5.19 | 6.51 | 70.89 | 1.27 | 11.70 |
| *n*-C29 | 4.89 | 6.13 | 77.02 | 2.72 | 12.50 |
| 11-/13-MeC27 | 3.65 | 4.57 | 81.59 | 7.29 | 0.00^†^ |
| *n*-C34 | 3.26 | 4.09 | 85.67 | 0.00^†^ | 6.52 |
| *n*-C35 | 1.97 | 2.48 | 88.15 | 0.00^†^ | 3.95 |
| 3,X-DiMeC28 | 1.69 | 2.11 | 90.26 | 3.37 | 0.00^†^ |
| 5,X-DiMeC27 | 1.51 | 1.89 | 92.15 | 3.01 | 0.00^†^ |
| 3-MeC27 | 1.14 | 1.43 | 93.57 | 2.27 | 0.00^†^ |
| *n*-C26 | 1.09 | 1.37 | 94.94 | 2.83 | 0.65 |
| *n*-C36 | 0.83 | 1.04 | 95.98 | 0.00^†^ | 1.67 |
| *n*-C28 | 0.65 | 0.82 | 96.80 | 2.86 | 3.46 |
| 5-MeC27 | 0.61 | 0.76 | 97.56 | 1.22 | 0.00^†^ |
| 3-MeC25 | 0.32 | 0.40 | 97.96 | 0.64 | 0.00^†^ |
| 5-MeC25 | 0.28 | 0.34 | 98.31 | 0.55 | 0.00^†^ |
| 12-/13-/14-MeC28 | 0.26 | 0.32 | 98.63 | 0.51 | 0.00^†^ |
| 5,11-DiMeC25 | 0.25 | 0.31 | 98.94 | 0.50 | 0.00^†^ |
| 10-/11-/12-/13-MeC26* | 0.24 | 0.30 | 99.24 | 0.48 | 0.00^†^ |
| 3-MeC26 | 0.19 | 0.23 | 99.47 | 0.37 | 0.00^†^ |
| 11-/13-MeC29* | 0.15 | 0.18 | 99.66 | 0.29 | 0.00^†^ |
| 4-MeC26 | 0.11 | 0.13 | 99.79 | 0.22 | 0.00^†^ |
| 4,14-DiMeC28 | 0.09 | 0.11 | 99.90 | 0.17 | 0.00^†^ |
| 11-/13-MeC25 | 0.08 | 0.10 | 100.00 | 0.16 | 0.00^†^ |
| 11,15-DiMeC27 | 0.00 | 0.00 | 100.00 | 0.00^†^ | 0.00^†^ |
| 3-MeC29 | 0.00 | 0.00 | 100.00 | 0.00^†^ | 0.00^†^ |
| 3-MeC28 | 0.00 | 0.00 | 100.00 | 0.00^†^ | 0.00^†^ |
| 4-MeC28 | 0.00 | 0.00 | 100.00 | 0.00^†^ | 0.00^†^ |

* Compounds with similar RI were pooled despite different positions of the methyl group

^†^ Compound is no component within the specific cuticular hydrocarbon profile of the two beetle species. For the SIMPER analysis, the value of this compound was determined as ‘0.00’

**Table S5** Mean amounts (ng ± SE larva^-1^) of cuticular hydrocarbons identified from T. confusum larval crude extracts before and after fractionation with 5Å-molecular sieves

|  |  |  |  | ***T. confusum* larval extracts^e^** | |
| --- | --- | --- | --- | --- | --- |
|  |  |  |  | Purified CHCs^f^ | Methyl alkanes^g^ |
| No^a^ | Compound^b^ | RI_cal_^c^ | RI_lit_^d^ | Mean ± SE (ng) | Mean ± SE (ng) |
| 1 | *n*-C25 | 2496 | 2500 | 12.19 ± 2.83 |  |
| 2 | 11-/13-MeC25 | 2530 | 2534 | 0.08 ± 0.03 | 0.02 ± 0.01 |
| 3 | 5-MeC25 | 2548 | 2550 | 0.04 ± 0.01 | 0.02 ± 0.01 |
| 4 | 3-MeC25 | 2569 | 2574 | 0.30 ± 0.07 | 0.12 ± 0.04 |
| 6 | *n*-C26 | 2596 | 2600 | 1.65 ± 0.41 |  |
| 7 | 10-/11-/12-MeC26 | 2629 | 2633 | 0.28 ± 0.08 | 0.17 ± 0.06 |
| 8 | 4-MeC26 | 2653 | 2658 | 0.07 ± 0.02 | 0.04 ± 0.02 |
| 10 | *n*-C27 | 2696 | 2700 | 28.62 ± 4.02 |  |
| 11 | 11-/13-MeC27 | 2727 | 2733 | 5.45 ± 0.88 | 4.07 ± 0.86 |
| 12 | 5-MeC27 | 2744 | 2750 | 1.43 ± 0.27 | 0.86 ± 0.25 |
| 14 | 3-MeC27 | 2767 | 2773 | 2.43 ± 0.34 | 1.46 ± 0.46 |
| 15 | 5,X-DiMeC27 | 2775 | 2781 | 0.75 ± 0.10 | 0.61 ± 0.08 |
| 16 | *n*-C28 | 2793 | 2800 | 3.12 ± 0.15 |  |
| 17 | 3,X-DiMeC28 | 2798 | 2807 | 0.85 ± 0.06 | 0.48 ± 0.07 |
| 18 | 12-/13-/14-MeC28 | 2827 | 2833 | 0.26 ± 0.06 | 0.20± 0.07 |
| 19 | 4-MeC28 | 2852 | 2856 | 0.13 ± 0.01 | 0.08 ± 0.02 |
| 20 | 3-MeC28 | 2870 | 2865 | 0.07 ± 0.02 | 0.02 ± 0.01 |
| 22 | *n*-C29 | 2894 | 2900 | 8.34 ± 0.40 |  |
| 23 | 11-/13-MeC29 | 2925 | 2931 | 0.82 ± 0.21 | 0.44 ± 0.14 |
| * | 5-MeC29 | 2942 | 2948 | 0.12 ± 0.07 | 0.14 ± 0.05 |
| 24 | 3-MeC29 | 2966 | 2978 | 0.27 ± 0.06 | 0.21 ± 0.08 |
| 25 | *n*-C30 | 2994 | 3000 | 0.48 ± 0.32 |  |
| 26 | *n*-C31 | 3090 | 3100 | 0.55 ± 0.42 |  |

^a^ Peak numbers refer to Figure 1b + c (main text); numbering of peaks relates to the numbering used in Table 2 (main text)

^b^ For the identification procedure see experimental part

^c^ RI_cal_ = Retention index calculated on a HP-5ms capillary column (30 m x 0.25 mm x 0.25 µm)

^d^ RI_lit_ = Retention index as reported for compounds analyzed on HP-5ms or similar columns in the database (<http://www.pherobase.com>/) and by Fürstenau and Hilker (2017) or others (peak 9 in Gerhardt et al. (2016)). The provided literature RI values of dimethyl alkanes (entries 15 and 17) refer to unambiguously identified compounds described as 5,13-diMeC27 and 3,13-diMeC27

^e^ Cuticular extracts of approx. 2000 *T. confusum* larvae were fractionated according to Bello et al. (2015)

^f^ For the preparation of samples see experimental part

^g^ For the preparation of samples see experimental part

^*^ 5-MeC29 was not found in our first GC-MS analysis of crude larval *T. confusum* extracts (see Table 2) but described earlier by Fürstenau and Hilker (2017)

**Table S6** Number of parasitized beetle larvae of potential host species (T. castaneum, T. confusum, T. destructor, O. surinamensis), which had been offered for 24 h to the parasitoid H. sylvanidis in oviposition bioassays, and the number of emerged H. sylvanidis adults after four weeks

| Host species | Number of parasitized beetle larvae  (after 24 h)^a^ | Number of emerged *H. sylvanidis* adults (after 4 weeks) |
| --- | --- | --- |
| *T. castaneum* | 29 | 26 |
| *T. confusum* | 27 | 25 |
| *T. destructor* | 23 | 22 |
| *O. surinamensis* | 0 | 0 |

^a^ We offered 40 live larvae of each host species (*N* = 40 per host species) for 24 h to *H. sylvanidis* females for oviposition


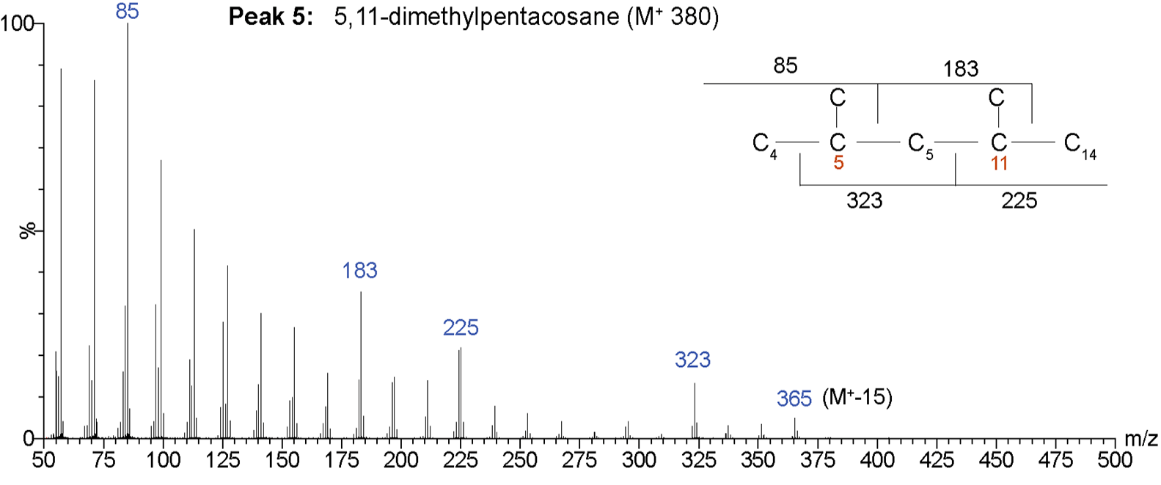


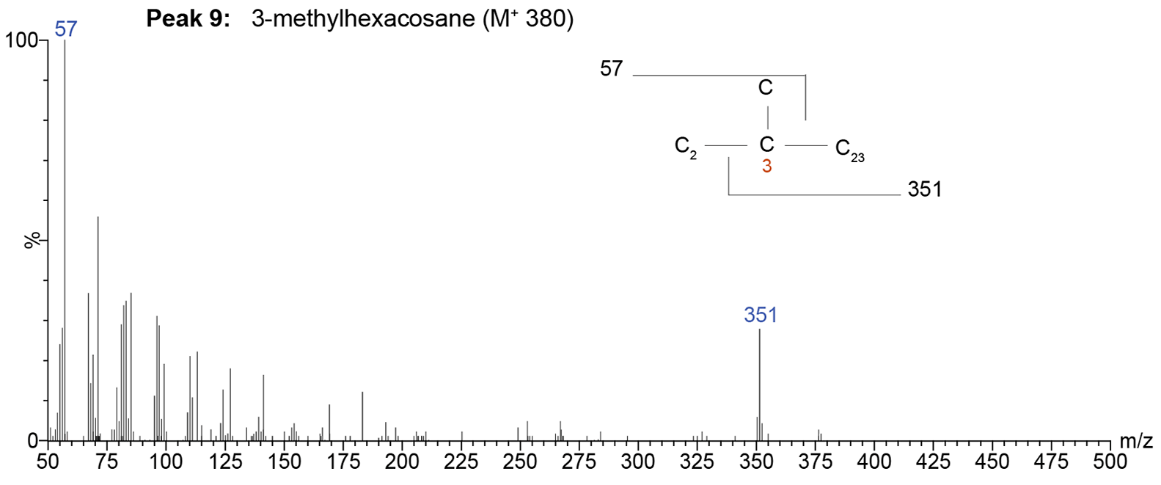


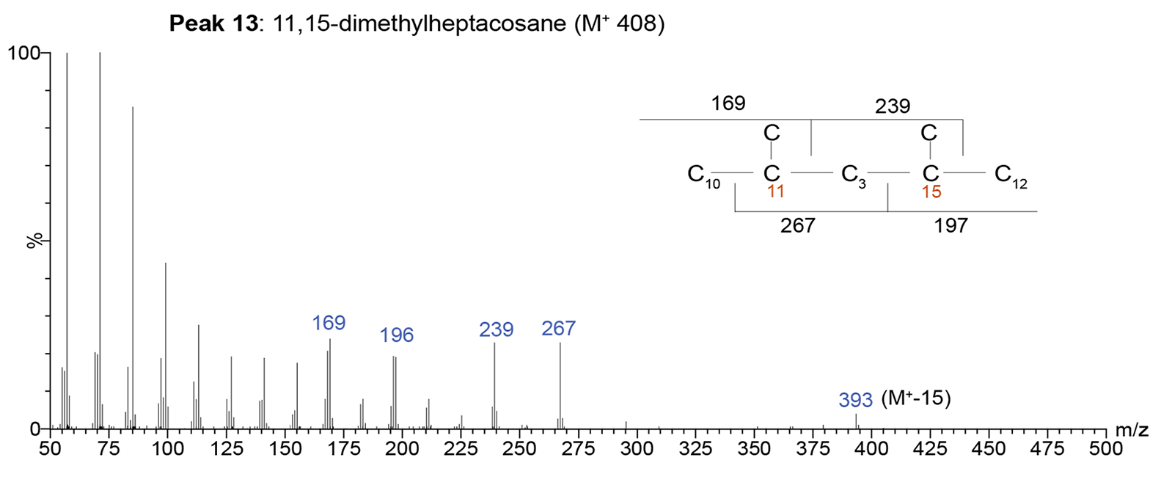


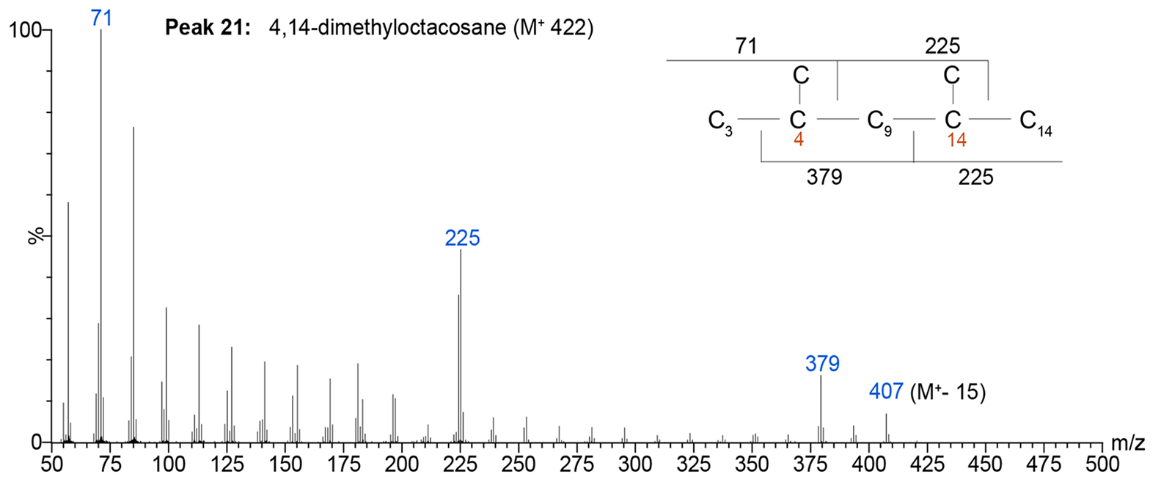


**Fig. S1** Mass spectra of monomethyl- and dimethyl branched CHCs identified from crude larval extracts of 4^th^ T. castaneum, T. confusum, and T. destructor. Previously, mass spectra and assignments of branching points (labelling diagnostic ions) of several methyl branched CHCs had been published (Fürstenau and Hilker, 2017). For these CHCs (peak 2, 3, 4, 7, 8, 11, 12, 14, 15, 17, 18, 19, 20, 23, 24 in Table 2, main text) positions of branching points were assigned based on characteristic fragmentation patterns displayed in the mass spectra and comparison with published data. Here we show mass spectra of CHCs (peak 5, 9, 13, and 21 in Table 2, main text), which were not detected previously. Diagnostic signals resulting from fragmentation at branching points and M^+^-15 fragments are labelled. Structure assignments are shown in the respective inserts


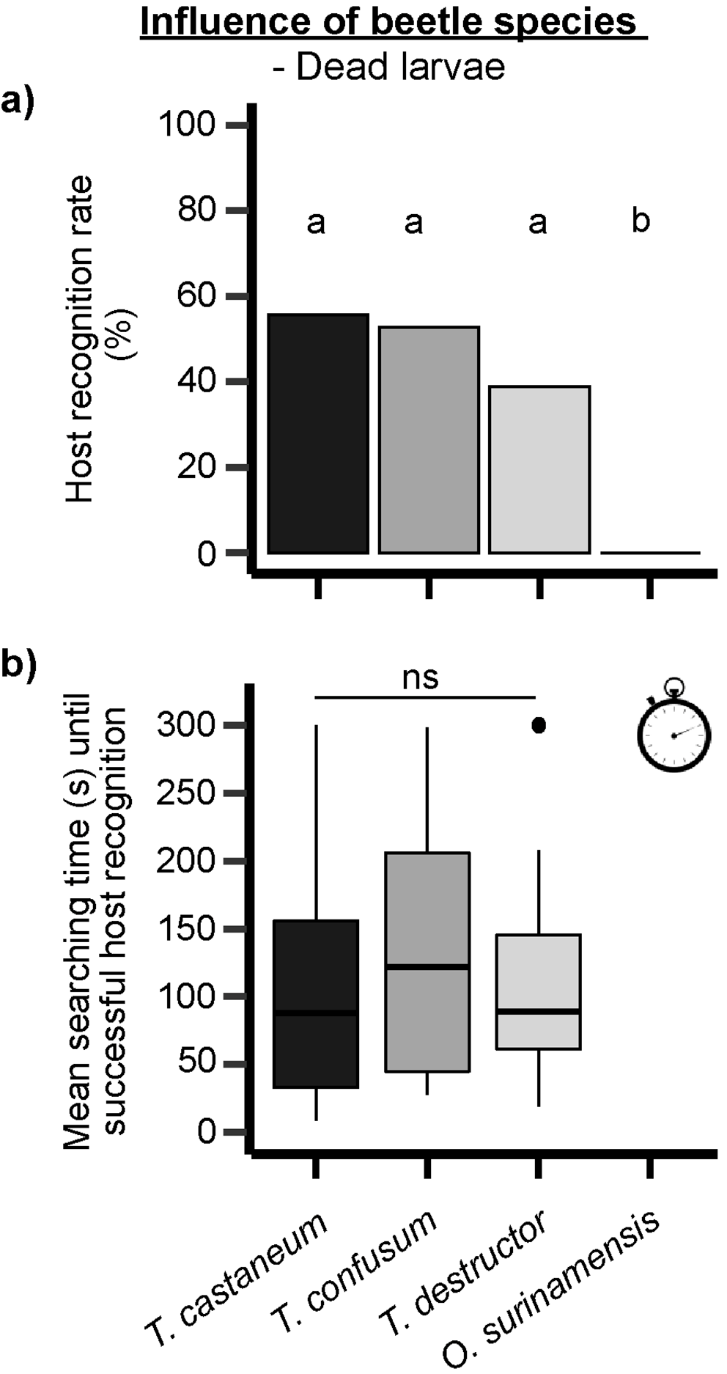


**Fig. S2** Contact bioassay: Behavioral responses of female H. sylvanidis ~~in~~ to freshly killed larvae of potential host species (T. castaneum, T. confusum, T. destructor and O. surinamensis; N = 36 per species, max. observation time = 300 s). **a)** Host recognition rate (100% ≙ 36 successful host recognition events per beetle species) was analyzed by the test for equality of proportions followed by pairwise comparison of proportions with Bonferroni-Holm correction. Different letters indicate significant differences at P < 0.05. **b)** Mean searching time until successful host recognition was analyzed for Tribolium spp. but not for O. surinamensis, as larvae of the latter species were rejected as hosts by the parasitoid. Statistical analysis was performed by Kruskal-Wallis test (ns = not significant, P > 0.05)


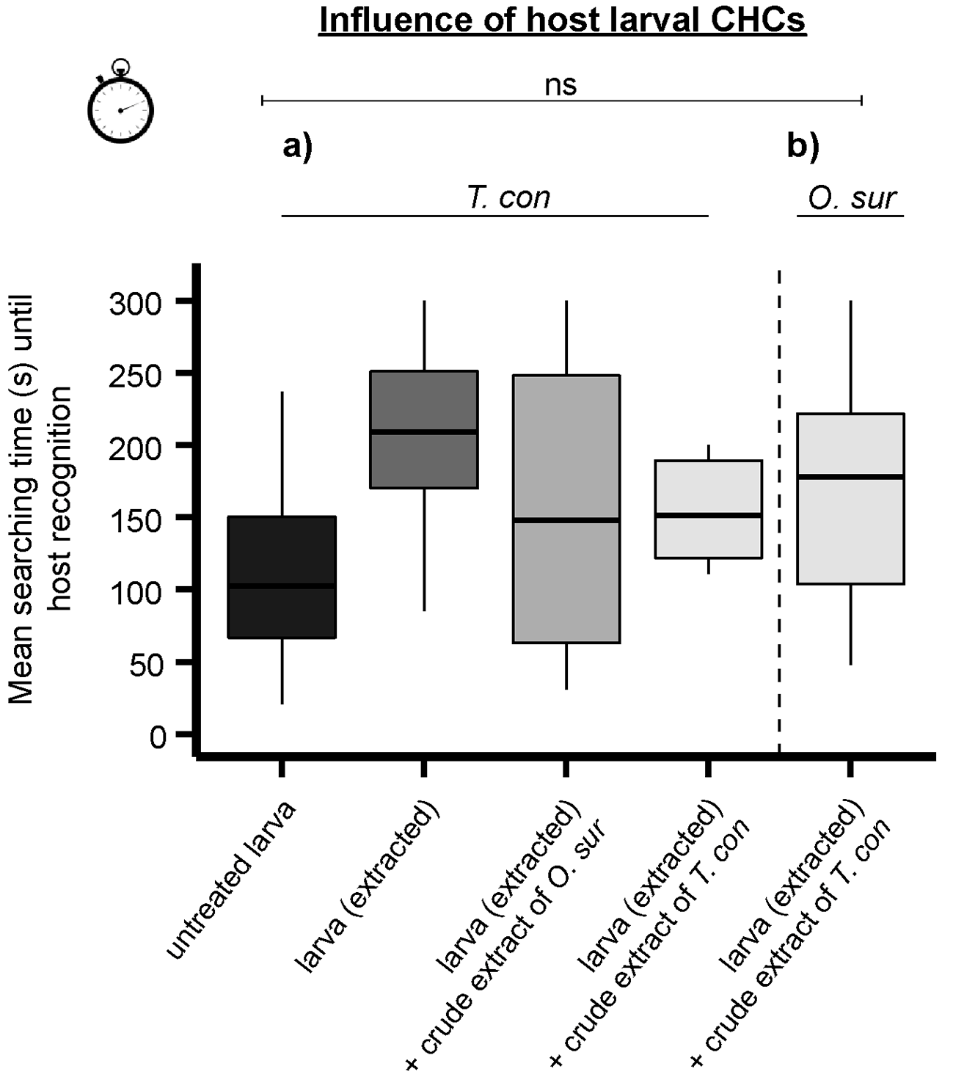


**Fig. S3** Mean searching time of female H. sylvanidis until successful host recognition of freshly killed **a)** T. confusum larvae (T. con) treated as follows (N = 30 per species and stimulus, max. observation time = 300 s): i) untreated, ii) extracted with n-hexane, iii) extracted with n-hexane and treated with a larval crude extract of O. surinamensis (O. sur, ½ LE), iv) extracted with n-hexane and treated with a larval crude extract of T. confusum (½ LE). **b)** As in the contact bioassays, offering O. surinamensis larvae only treatment iv (dead larvae (extracted) + larval crude extract of T. con,) elicited successful host recognition behavior in H. sylvanidis (see Fig. 4, main text), only for this treatment the mean searching time could be determined and statistically compared by Kruskal-Wallis test (ns = not significant, P > 0.05)

~~
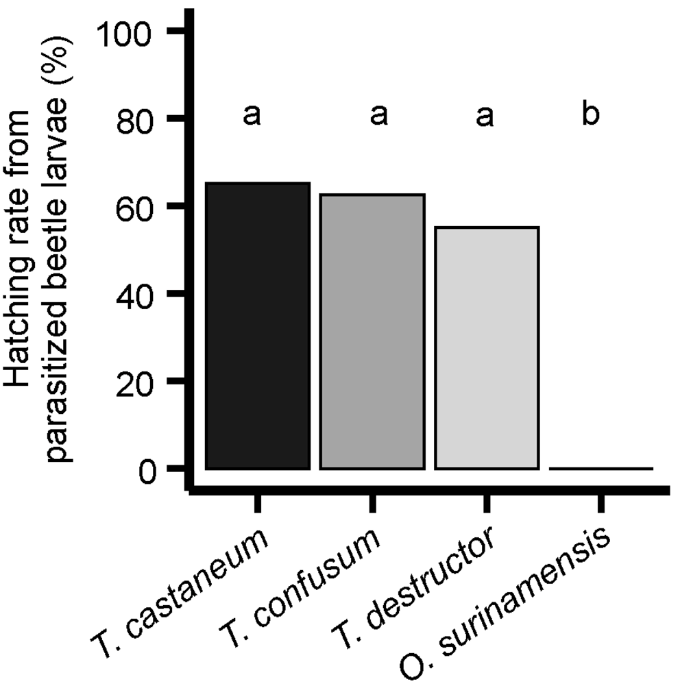
~~

**Fig. S4** Hatching rate of adult *H. sylvanidis* from beetle larvae which had been offered as potential host species (*T. castaneum*, *T. confusum*, *T. destructor* and *O. surinamensis*, *N* = 40 per species) to female parasitoids for 24 h in oviposition bioassays. Data were analyzed by the test for equality of proportions followed by pairwise comparison of proportions with *Bonferroni-Holm* correction. Different letters indicate significant differences at *P* < 0.05
